# Pentoxifylline Alone or in Combination with Gentamicin or Vancomycin Inhibits Live Microbe-Induced Proinflammatory Cytokine Production in Human Cord Blood and Cord Blood Monocytes *In Vitro*

**DOI:** 10.1128/AAC.01462-18

**Published:** 2018-11-26

**Authors:** Esther M. Speer, Elizabeth Diago-Navarro, Lukasz S. Ozog, David J. Dowling, Wei Hou, Mahnoor Raheel, Bettina C. Fries, Ofer Levy

**Affiliations:** a Department of Pediatrics, Stony Brook University School of Medicine, Stony Brook, New York, USA; b Division of Infectious Diseases, Department of Medicine, Stony Brook University School of Medicine, Stony Brook, New York, USA; c Precision Vaccine Program, Division of Infectious Diseases, Boston Children’s Hospital, Boston, Massachusetts, USA; d Harvard Medical School, Boston, Massachusetts, USA; e Family, Population, and Preventive Medicine Department, Stony Brook University School of Medicine, Stony Brook, New York, USA; f Broad Institute of MIT and Harvard, Cambridge, Massachusetts, USA

**Keywords:** *Escherichia coli*, *Staphylococcus aureus*, antimicrobial agents, coagulase-negative staphylococci, cord blood, cytokines, human newborn, inflammatory, neonatal sepsis, pentoxifylline

## Abstract

Neonatal sepsis and its accompanying inflammatory response contribute to substantial morbidity and mortality. Pentoxifylline (PTX), a phosphodiesterase inhibitor which suppresses transcription and production of proinflammatory cytokines, is a candidate adjunctive therapy for newborn sepsis. We hypothesized that PTX decreases live microbe-induced inflammatory cytokine production in newborn blood. Cord blood was stimulated with live microorganisms commonly encountered in newborn sepsis (Escherichia coli, Staphylococcus aureus, Staphylococcus epidermidis, or Candida albicans) and simultaneously treated with antimicrobial agents (gentamicin, vancomycin, or amphotericin B) and/or clinically relevant concentrations of PTX. Microbial colony counts were enumerated by plating, supernatant cytokines were measured by multiplex assay, intracellular cytokines and signaling molecules were measured by flow cytometry, and mRNA levels were measured by quantitative reverse transcription-PCR. PTX inhibited concentration-dependent E. coli-, S. aureus-, S. epidermidis-, and C. albicans-induced tumor necrosis factor (TNF) and E. coli-induced interleukin-1β (IL-1β) production in whole blood, with greater suppression of proinflammatory cytokines in combination with antimicrobial agents. Likewise, PTX suppressed E. coli-induced monocytic TNF and IL-1β, whereby combined PTX and gentamicin led to significantly greater reduction of TNF and IL-1β. The anti-inflammatory effect of PTX on microbe-induced proinflammatory cytokine production was accompanied by inhibition of *TNF* mRNA expression and was achieved without suppressing the production of the anti-inflammatory IL-10. Of note, microbial colony counts in newborn blood were not increased by PTX. Our findings demonstrated that PTX inhibited microbe-induced proinflammatory cytokine production, especially when combined with antimicrobial agents, without enhancing microbial proliferation in human cord blood *in vitro*, thus supporting its utility as candidate adjunctive agent for newborn sepsis.

## Abstract (precis)

Neonatal sepsis and its accompanying inflammatory response contribute to substantial morbidity and mortality. Pentoxifylline (PTX), a phosphodiesterase inhibitor which suppresses transcription and production of proinflammatory cytokines, is a candidate adjunctive therapy for newborn sepsis.

## INTRODUCTION

An estimated 1 million newborns die annually worldwide during the first 4 weeks of life due to severe infections such as sepsis and pneumonia (1), with a particularly high risk among preterm newborns, who represent ∼11% of all live births globally (1, 2). The neonatal inflammatory response to intrauterine or postnatal infections is mediated in part via pattern recognition receptors (PRRs) (3, 4). While important to host defense, this inflammatory response is associated with increased mortality and morbidity, as manifested by the acute systemic inflammatory response syndrome characteristic for severe sepsis and end-organ damage, including perinatal brain injury (5–7). Hence, appropriately formulated and timed anti-inflammatory agents, when given together with antimicrobial agents, may be of benefit as adjunctive newborn sepsis therapy. However, corticosteroids are associated with significant side effects and are generally not recommended for neonatal sepsis (8). Indeed, effective alternative anti-inflammatory therapies suitable for neonates are lacking to date.

The phosphodiesterase inhibitor pentoxifylline (PTX), which increases intracellular cyclic AMP (cAMP) and decreases transcription of proinflammatory mediators, including tumor necrosis factor (TNF) (9, 10), is a candidate neonatal anti-inflammatory agent. PTX is currently under study as an adjunctive therapy for newborn sepsis and necrotizing enterocolitis (11, 12). PTX decreased Toll-like receptor (TLR)-mediated proinflammatory cytokine production with higher efficacy and potency in newborn cord blood compared to adult blood (13). Furthermore, PTX combined with dexamethasone or azithromycin synergistically inhibited TLR- and inflammasome-mediated proinflammatory cytokine production in human newborn and adult blood *in vitro*, thus potentially limiting drug exposure and toxicity (14).

Although the effects of PTX in reducing inflammatory responses to pure PRR agonists are promising, thus far little is known regarding the anti-inflammatory effects of PTX on activation of whole human neonatal blood and blood-derived leukocytes by live bacterial pathogens. Indeed, the effects of isolated pattern recognition receptor agonists are not reflective of the stimulation of the innate immune system by live pathogens. The innate immune system detects viability-associated pathogen-associated molecular patterns such as bacterial mRNA resulting in greater inflammatory responses (15–17). Particle size can also affect innate immune responses (18), and PRRs such as TLR8 can recognize live bacteria, eliciting a unique and more robust innate and adaptive immune response (19). Candidate adjunct anti-inflammatory agents intended to suppress sepsis-induced acute systemic inflammatory responses should be evaluated for their ability to inhibit proinflammatory immune responses induced by relevant live microorganisms. On the other hand, pharmacological suppression of innate inflammatory immune responses could potentially compromise host antimicrobial defense mechanisms, thus allowing for increased replication of invading microorganisms. Based on the cytokine-inhibiting actions of PTX in conjunction with potential increased bacterial clearance (20), we hypothesized that PTX decreases live microbe-induced proinflammatory cytokine production in newborn blood and blood-derived monocytes without enhancing microbial proliferation.

In order to optimally mirror physiological conditions encountered in neonatal sepsis *in vitro*, we stimulated whole newborn cord blood, which contains a multitude of anti-inflammatory humoral and cellular factors (21, 22), with live microorganisms that are commonly encountered in newborn sepsis (23), including Staphylococcus epidermidis, Staphylococcus aureus, Escherichia coli, and Candida albicans, and treated blood samples with clinically relevant concentrations of conventional antimicrobial agents and PTX (24, 25). We determined the effects of PTX alone and combined with antimicrobial agents on pro- and anti-inflammatory cytokine transcription and protein production in newborn cord blood and cord blood-derived monocytes, as well as their effects on the expression of PRRs and signaling molecules. PTX inhibited E. coli-, S. aureus-, S. epidermidis-, and C. albicans-induced TNF and E. coli-induced interleukin-1β (IL-1β) production in whole-blood cultures, and E. coli-induced TNF and IL-1β, as well as S. epidermidis-induced IL-1β, but not TNF in cord blood monocytes, without decreasing IL-6 and IL-10. At the transcriptional level, PTX decreased E. coli-, S. aureus-, and C. albicans-, but not S. epidermidis-induced *TNF* mRNA expression, inhibited C. albicans-induced *IL1B* mRNA but increased C. albicans-induced *IL6* mRNA expression. PTX combined with amphotericin B (AMB) but not PTX alone increased C. albicans-induced *IL10* mRNA. Enhanced suppression of proinflammatory cytokines, but no effect on pathogen burden, was observed *in vitro* when PTX was administered in combination with antimicrobial agents, supporting the potential utility of PTX as an adjunctive anti-inflammatory agent for newborn sepsis.

## RESULTS

### PTX did not alter microbial colony counts in newborn blood *in vitro*.

**FIG 1:**
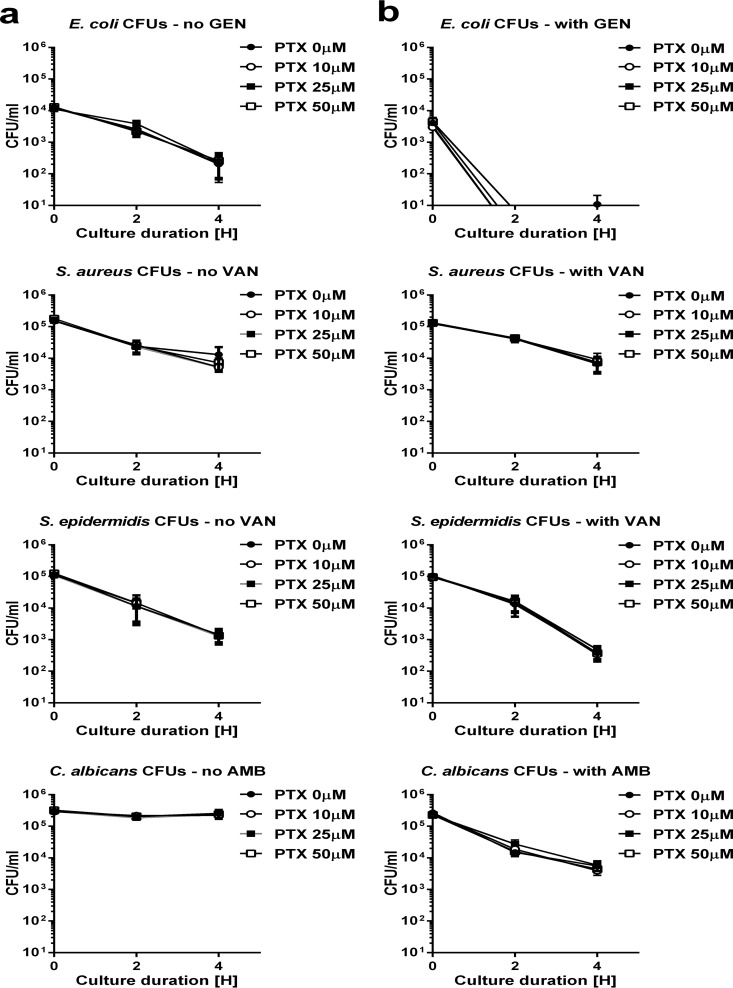
PTX did not enhance microbial proliferation in newborn cord blood. Cord blood samples (*n* = 10 to 13) were treated with vehicle control or increasing concentrations (10 to 50 µM) of PTX in the absence (a) or presence (b) of antimicrobial agents and simultaneously inoculated with live E. coli (10^4^ CFU/ml), S. aureus (10^5^ CFU/ml), S. epidermidis (10^5^ CFU/ml), or C. albicans (10^5^ CFU/ml). After 0, 2, and 4 h in culture (5% CO_2_, 37°C), blood samples were plated with serial dilutions, and the CFU/ml values were determined.

Immunomodulatory agents such as PTX could potentially modify the replication of microorganisms, which would prohibit their clinical use. We therefore determined the growth pattern of the studied microorganisms in newborn cord blood treated with increasing concentrations of PTX and with or without the addition of antimicrobial agents. The CFU of all tested microorganisms in the presence or absence of susceptible antimicrobial agents, except for C. albicans without the addition of AMB, decreased with increasing duration of culture in newborn blood *in vitro* (Fig. 1). E. coli CFU decreased the most rapidly in blood samples treated with gentamicin (GEN), and were mostly undetectable after 4 h. Addition of the anti-inflammatory agent PTX did not enhance microbial proliferation of E. coli, S. aureus, S. epidermidis, or C. albicans (Fig. 1).

### VAN, GEN, and AMB did not exert any anti-inflammatory effects in the absence of live microorganisms in newborn blood.

Since vancomycin (VAN), GEN, and AMB all demonstrated significant inhibition of microbe-induced proinflammatory cytokine production in newborn cord blood, as described in more detail below, we tested whether these agents exhibit intrinsic anti-inflammatory properties. We therefore stimulated and cultured cord blood samples with purified TLR2 (peptidoglycan [PGN] from S. aureus) and TLR4 (lipopolysaccharide [LPS] from E. coli) agonists in the presence or absence of these antimicrobial agents and measured cytokine responses in culture supernatants. As shown in Fig. S1 in the supplemental material, none of the tested antimicrobial agents modified the production of TLR-mediated pro- and anti-inflammatory cytokine production in cord blood. These observations suggest that antimicrobial-induced alterations of live E. coli, S. aureus, S. epidermidis, and C. albicans contributed to the observed reduction of proinflammatory cytokine production in newborn cord blood.

### PTX inhibited *E. coli*-induced proinflammatory cytokine production in newborn blood.

We first determined the baseline cytokine-inducing patterns in human cord blood by the live microorganisms used in this study in the absence of anti-inflammatory and antimicrobial agents. As demonstrated in Fig. S2, all live microorganisms induced pro- and anti-inflammatory cytokine production in newborn cord blood. However, even at a 10-fold-lower inoculum (E. coli, 10^4^ CFU/ml; all other microorganisms, 10^5^ CFU/ml), E. coli induced significantly greater production of TNF, IL-1β, and IL-10 compared to S. epidermidis and C. albicans, as well as greater production of IL-1β and IL-10 compared to S. aureus.

**FIG 2:**
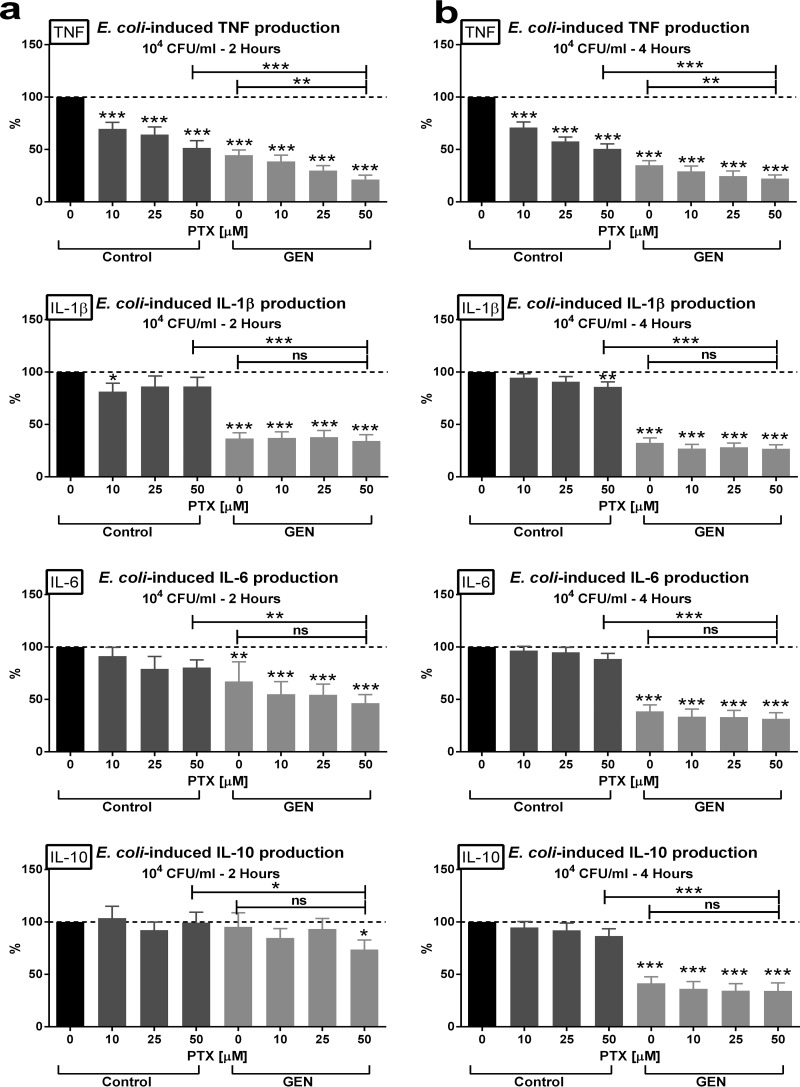
Enhanced inhibition of E. coli-induced TNF production in newborn cord blood by the combination of PTX and GEN. Cord blood samples (*n* = 10 to 13) were stimulated with live E. coli at 10^4^ CFU/ml; simultaneously treated with vehicle control, PTX, GEN, or PTX+GEN at the indicated concentrations; and cultured for 2 h (a) or 4 h (b) in 5% CO_2_ at 37°C. Cytokine concentrations of treated samples were expressed as a percentage compared to untreated samples stimulated with E. coli alone, which were defined as 100%. Significant differences between treated versus untreated samples and between samples treated with single (antimicrobial or anti-inflammatory) agents versus combination treatment are indicated (*, *P* < 0.05; **, *P* < 0.01; ***, *P* < 0.001).

PTX alone, when added at clinically relevant concentrations (10 to 50 µM) to newborn cord blood, inhibited E. coli-induced TNF and, at the highest concentration, also inhibited IL-1β production while preserving the proresolution and anti-inflammatory cytokines IL-6 and IL-10 (Fig. 2). In contrast, GEN suppressed the production of all tested cytokines. PTX plus GEN in combination (PTX+GEN) exerted greater inhibition of E. coli-induced TNF production than either agent alone (50.6 ± 4.7% in PTX-treated, 35.0 ± 4.3% in GEN-treated, and 22.1 ± 3.6% in PTX+GEN-treated cord blood; reference value, 100% in untreated cord blood), indicating potentially greater anti-inflammatory efficacy for this combination.

### PTX (and PTX + other antimicrobial agents) selectively inhibited *S. aureus*-, *S. epidermidis*-, and *C. albicans*-induced TNF production in whole newborn blood.

**FIG 3:**
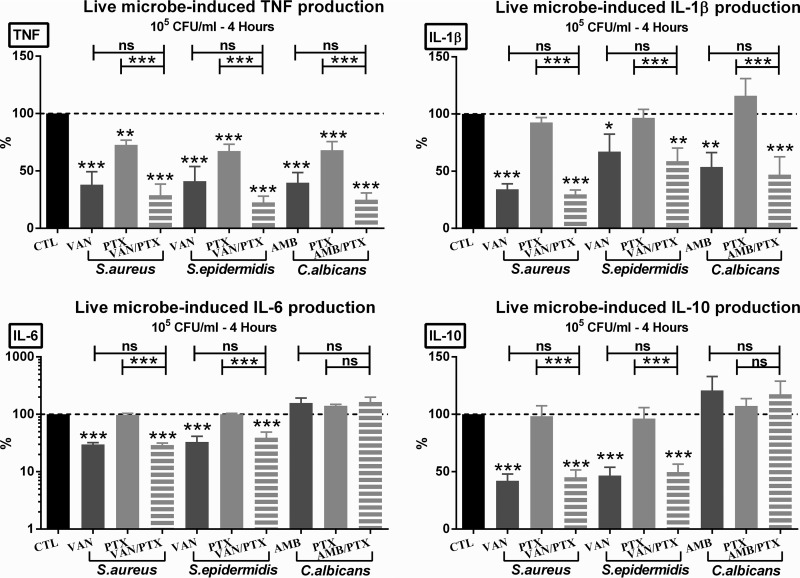
PTX and antimicrobial agents alone or combined suppressed live microbial-induced TNF production. Cord blood samples (*n* = 10 to 13) were stimulated with 10^5^ CFU/ml of live S. aureus, S. epidermidis, or C. albicans and simultaneously treated with vehicle control, 50 µM PTX, antimicrobial agents, or PTX + other antimicrobial agents and then cultured for 4 h in 5% CO_2_ at 37°C. Cytokine concentrations of treated samples were expressed as a percentage compared to untreated samples stimulated with microorganisms alone, which were defined as 100%. Significant differences between treated versus untreated samples and between samples treated with single (PTX or antimicrobial) agents versus combination treatment are indicated (*, *P* < 0.05; **, *P* < 0.01; ***, *P* < 0.001).

In order to mirror the effects of PTX and antimicrobial agents on cytokine production in newborn sepsis, we tested the effects of this anti-inflammatory agent in the presence or absence of antimicrobial agents on live microorganism-induced cytokine production in newborn cord blood. PTX alone or PTX combined with VAN or AMB suppressed the production of live microbe-induced TNF in newborn cord blood (Fig. 3), with significantly greater inhibition of TNF production in response to PTX plus other antimicrobial agents compared to PTX alone. VAN, in the presence or absence of PTX, inhibited the production of all microbe-induced pro- and anti-inflammatory cytokines tested (Fig. 3). PTX alone was associated with a minor reduction in E. coli-induced IL-1β as described above but did not decrease the production of IL-1β, IL-6, and IL-10 induced by the other live microorganisms. Likewise, AMB alone or combined with PTX inhibited live C. albicans-induced TNF production, in addition to a minor suppressive effect on IL-1β, but it did not decrease C. albicans-induced IL-6 and IL-10 production (Fig. 3).

### PTX, GEN, and PTX+GEN inhibited live *E. coli*-induced proinflammatory intracellular cytokines in cord blood monocytes.

**FIG 4:**
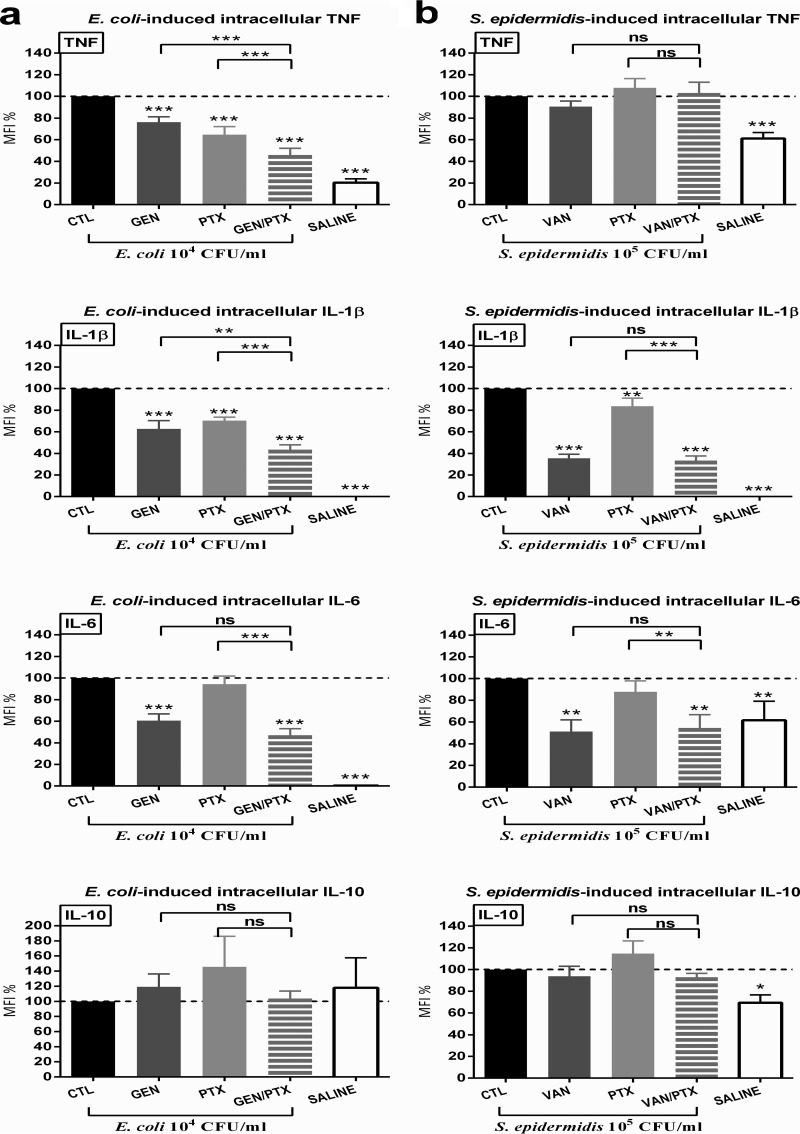
PTX, GEN, and combined PTX+GEN inhibited live E. coli-induced proinflammatory intracellular cytokines in human CD14^+^ CD45^+^ cord blood monocytes. Whole cord blood (*n* = 10) was stimulated with live E. coli (10^4^ CFU/ml) (a) or S. epidermidis (10^5^ CFU/ml) (b), simultaneously treated with 200 μM PTX and/or antibiotics or vehicle control, and cultured for 4 h in 5% CO_2_ at 37°C. Samples underwent multicolor staining with monoclonal antibodies for flow cytometric measurements, as described in Materials and Methods. MFI values of treated samples are expressed as a percentage compared to untreated samples stimulated with microorganisms alone, which were defined as 100%. Significant differences between treated versus untreated samples and between samples treated with single (PTX or antimicrobial) agents versus combination treatment are indicated (*, *P* < 0.05; **, *P* < 0.01; ***, *P* < 0.001).

Next, we tested the effects of PTX and antibiotics on microbe-induced intracellular cytokines in cord blood monocytes. This was done because monocytes are the predominant producers of cytokines in response to microbial and inflammatory signaling. E. coli and S. epidermidis both induced a robust response with significantly increased pro- and anti-inflammatory intracellular cytokine concentrations (Fig. 4 and Fig. S3). Comparable to the findings in whole blood culture supernatants, E. coli induced significantly greater increases in intracellular TNF, IL-1β, and IL-6 concentrations, despite the 10-fold-lower bacterial inoculum used in these *in vitro* experiments (see Fig. S3). In contrast, E. coli and S. epidermidis induced comparable levels of intracellular IL-10 in untreated newborn monocytes (Fig. S3).

As demonstrated in Fig. 4, PTX, GEN, and PTX+GEN decreased E. coli-induced intracellular TNF and IL-1β, whereby the drug combination exerted significantly greater cytokine suppressive effects compared to the single agents. VAN or GEN alone decreased E. coli-induced intracellular TNF, IL-1β, and IL-6 and S. epidermidis-induced IL-1β and IL-6 in newborn monocytes. PTX alone inhibited E. coli-induced TNF and IL-1β and S. epidermidis-induced IL-1β without suppressing intracellular concentrations of microbe-induced proresolution and anti-inflammatory IL-6 and IL-10. Whereas PTX exerted only a minor decrease in magnitude of E. coli-induced IL-1β concentrations in blood culture supernatants (Fig. 2), this agent significantly suppressed intracellular concentrations of IL-1β in cord blood monocytes (Fig. 4), suggesting a potential greater sensitivity of newborn monocytes toward the anti-inflammatory effects of PTX.

### Effect of PTX on live microbe-induced TLR surface expression and intracellular signaling molecules in cord blood monocytes.

**FIG 5:**
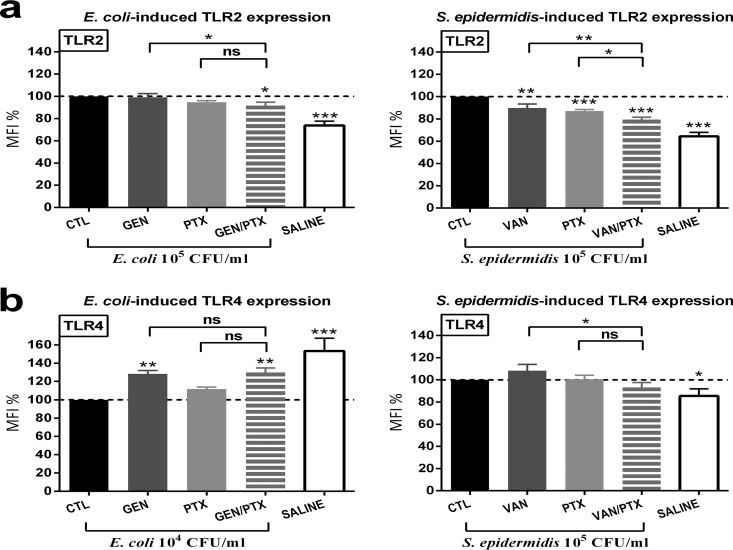
PTX and/or other antimicrobial agent inhibited live E. coli- and S. epidermidis-induced TLR2 and increased TLR4 surface receptor expression in CD14^+^ CD45^+^ cord blood monocytes. Cord blood samples (*n* = 10) were stimulated with live E. coli (10^4^ CFU/ml) or S. epidermidis (10^5^ CFU/ml), simultaneously treated with 200 μM PTX and/or antibiotics or vehicle control, and cultured for 4 h in 5% CO_2_ at 37°C. Samples underwent multicolor staining for flow cytometry with monoclonal antibodies against surface receptors, as described in Materials and Methods. MFI values of treated samples are expressed as a percentage compared to untreated samples stimulated with microorganisms alone, which were defined as 100%. (a and b) TLR2 (a) and TLR4 (b) surface receptor expression. Significant differences between treated versus untreated samples and between samples treated with single (PTX or antimicrobial) agents versus combination treatment are indicated (*, *P* < 0.05; **, *P* < 0.01; ***, *P* < 0.001).

In order to determine whether differences in TLR surface expression in response to PTX and/or antibiotics could explain the observed differences in the inhibition of E. coli-induced versus S. epidermidis-induced cytokine production, we measured the expression of TLR2 and TLR4 in cord blood monocytes. Saline control samples included in these experiments showed the baseline TLR surface expression in the absence of microbial stimulation. PTX, VAN, and PTX+VAN decreased S. epidermidis-induced TLR2 surface receptor expression, with significantly greater suppression for the drug combination compared to the single agents, whereas only the combined PTX+GEN showed a minimal suppressive effect on E. coli-induced TLR2 surface receptor suppression (Fig. 5a). GEN with or without PTX enhanced E. coli-induced TLR4 surface receptor expression toward baseline levels, while PTX alone did not show any effect on TLR4 surface receptor expression (Fig. 5b).

To clarify whether PTX exerted its anti-inflammatory effects at least in part through inhibition of the two major inflammatory signaling pathways, mitogen activated protein kinase (MAPK) or nuclear factor κB, we measured the phosphorylation of MAPK and the degradation of inhibitory κB (IκB). E. coli, as opposed to S. epidermidis, robustly induced p38 MAPK phosphorylation and IκBα degradation (Fig. S4). PTX, however, did not modify the activation of these intracellular signaling molecules (Fig. S4), suggesting that these pathways do not play a role in the anti-inflammatory response to PTX.

### PTX and/or antimicrobial agents decreased live microorganism-induced mRNA expression of proinflammatory cytokine genes without inhibiting *IL10* mRNA in newborn cord blood.

**FIG 6:**
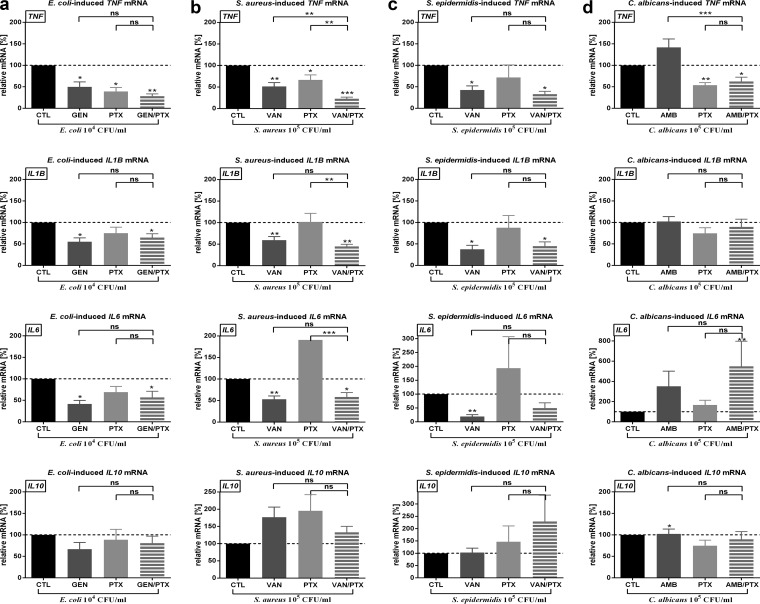
PTX and/or antimicrobial agents inhibited live microorganisms-induced *TNF* mRNA expression in whole newborn cord blood. Cord blood (*n* = 10) was stimulated with live microorganisms—E. coli at 10^4^ CFU/ml (a), S. aureus at 10^5^ CFU/ml (b), S. epidermidis at 10^5^ CFU/ml (c), or C. albicans at 10^5^ CFU/ml (d)—and simultaneously treated with 200 µM PTX and/or antimicrobial agents or vehicle control and then cultured for 4 h in 5% CO_2_ at 37°C. Relative mRNA expressions of samples stimulated with microorganisms and treated with PTX and/or antimicrobial agents are calculated from ΔΔ*C_T_* values and normalized to microbial stimulation alone, which was defined as 100%. Significant differences between ΔΔ*C_T_* values of treated versus untreated samples and between samples treated with single (PTX or antimicrobial) agents versus combination treatment are indicated (*, *P* < 0.05; **, *P* < 0.01; ***, *P* < 0.001).

Since PTX exerts its anti-inflammatory effects through transcriptional regulation of proinflammatory cytokines, we measured mRNA expression in cord blood samples that were stimulated with live microorganisms and treated with PTX and/or antimicrobial agents. PTX (and PTX plus other antimicrobial agents) decreased live E. coli-, S. aureus-, and C. albicans-induced *TNF* mRNA expression in whole cord blood, with significantly greater suppression of the *TNF* gene for the use of combined agents (PTX + other antimicrobial) versus the use of antimicrobial agents alone (Fig. 6 and Tables S1 and S2). For example, S. aureus-induced relative *TNF* mRNA expression was reduced from a reference value of 100% (untreated cord blood) to 44.1% (95% confidence interval = 30.2 to 64.5) in VAN-treated, 56.1% (37.5 to 84.0) in PTX-treated, and 19.5% (13.3 to 28.6) in VAN+PTX-treated newborn blood. Likewise, E. coli-induced relative *TNF* mRNA was reduced from 100% (untreated blood) to 33.1% (16.6 to 66.0) in GEN-treated, 23.1% (10.6 to 50.0) in PTX-treated, and 19.8% (10.6 to 36.9) in GEN+PTX-treated cord blood samples, respectively. The PTX-mediated inhibition of *TNF* mRNA expression was sustained over 1 and 2 h of culture. S. epidermidis-induced *TNF* mRNA, on the other hand, was reduced by VAN with or without PTX but not by PTX alone. In contrast, GEN or VAN, whether alone or in combination with PTX, suppressed microbe-induced mRNA expression of *TNF*, *IL1B*, and *IL6*. Whereas PTX alone decreased C. albicans-induced *TNF* and *IL1B* mRNA and PTX+AMB also inhibited C. albicans-induced *TNF* mRNA expression, AMB alone did not modify gene expression of these cytokines (Fig. 6 and Table S1). PTX, AMB, and PTX+AMB enhanced C. albicans-induced mRNA expression of the proresolution cytokine *IL6*, whereby the combination of agents led to a greater increase in gene expression compared to PTX alone (Tables S1 and S2). Except for enhanced expression of C. albicans-induced *IL10* mRNA by AMB, microbe-induced *IL10* gene expression was not modified by PTX or any of the antimicrobial agents tested (Fig. 6 and Table S1). Likewise, PTX and/or other antimicrobial agents did not significantly change live microbe-induced expression of genes encoding TLR2 and TLR4, nuclear factor κB p65 and IκBα, dual specificity phosphatase 1, caspase 1, NACHT, LRR, and PYD domain-containing protein 3 (Tables S1 and S2).

### PTX inhibited TLR2- and TLR4-mediated proinflammatory cytokine production in whole cord blood and cord blood monocytes.

**FIG 7:**
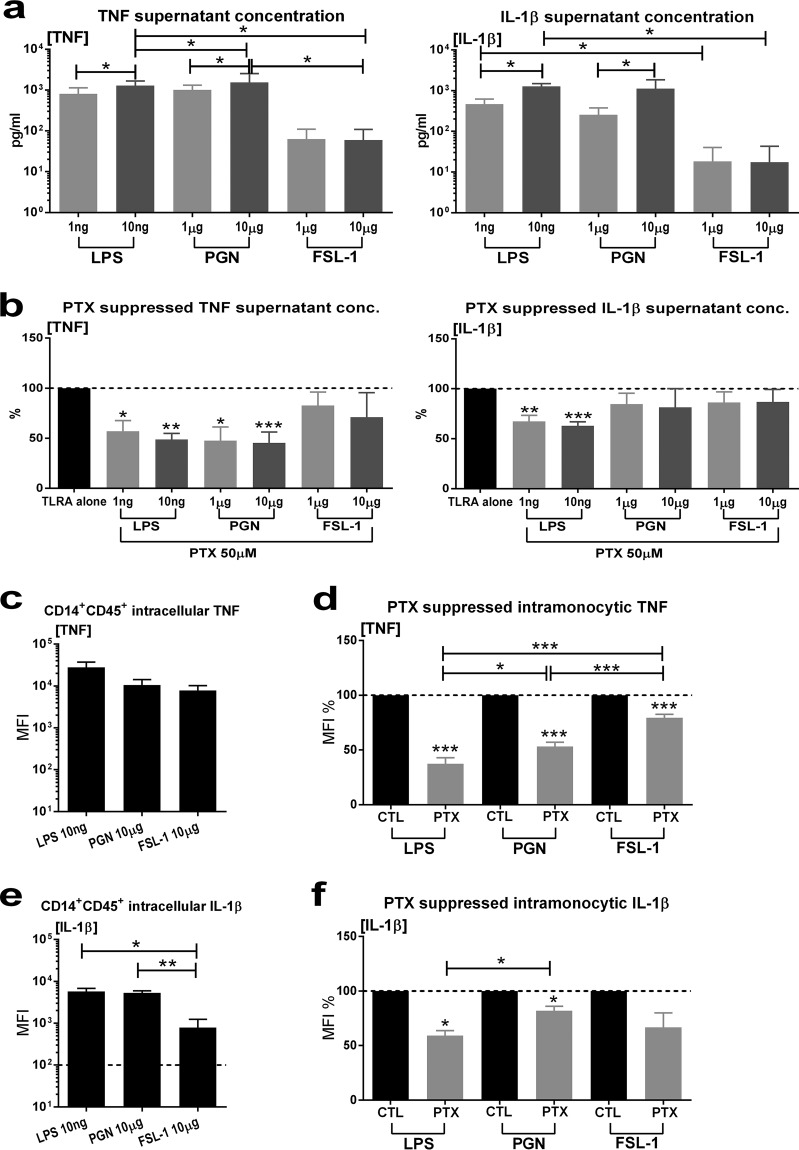
PTX inhibited TLR2- and TLR4-mediated proinflammatory cytokine production in newborn cord blood and cord blood monocytes. Cord blood (*n* = 5) was stimulated with purified TLR2 (PGN and FSL-1) or TLR4 (LPS) agonists and simultaneously treated with PTX or vehicle control. (a) TLR2 and TLR4 agonist-induced proinflammatory cytokines in cord blood culture supernatants. (b) Effect of PTX on TLR2 and TLR4 agonist-induced TNF and IL-1β in cord blood culture supernatants. (c) TLR2 and TLR4 agonist-induced intracellular TNF in cord blood monocytes. (d) Inhibition of TLR2- and TLR4-mediated intramonocytic TNF by PTX. (e) TLR2 and TLR4 agonist-induced intracellular IL-1β in cord blood monocytes. (f) Inhibition of TLR2 and TLR4 agonist-induced intramonocytic IL-1β by PTX. Cytokine concentrations and MFI values of treated samples are expressed as percentages compared to untreated samples stimulated with TLR agonists alone, which were defined as 100%. Significant differences between treated versus untreated samples and between samples stimulated with TLR2 versus TLR4 agonists are indicated (*, *P* < 0.05; **, *P* < 0.01; ***, *P* < 0.001).

Since PTX appeared to inhibit E. coli-induced proinflammatory cytokine production in cord blood monocytes as well as E. coli-induced *TNF* mRNA expression in whole blood but not in S. epidermidis-exposed newborn cord blood and cord blood monocytes (Fig. 4 and 6), we explored the anti-inflammatory effects of PTX on TLR2-mediated versus TLR4-mediated cytokine production in whole cord blood and cord blood monocytes. Purified TLR2 (PGN and FSL-1, a synthetic diacylated lipoprotein) and TLR4 agonists (Fig. 7a) induced a concentration-dependent increased production of TNF and IL-1β in whole cord blood samples. LPS and PGN induced greater increases in cytokine production compared to FSL-1. PTX suppressed the production of PGN- and LPS-induced but not of FSL-1-induced TNF and IL-1β production in whole cord blood, regardless of the TLR agonist concentration added to blood samples, indicating that the anti-inflammatory mechanisms of action of PTX may be independent of the TLR pathway engaged, as well as the level of inflammation induced (Fig. 7b). Analogous to whole cord blood, stimulation of blood samples with TLR2 and TLR4 agonists increased the intracellular TNF and IL-1β concentrations in CD14^+^ CD45^+^ cord blood monocytes (Fig. 7c and e) with greater cytokine responses in LPS- and PGN-exposed monocytes compared to FSL-1-exposed monocytes. PTX markedly suppressed intramonocytic TNF in both TLR2- and TLR4-stimulated cord blood monocytes (Fig. 7d), albeit to a lesser magnitude in FSL-1-stimulated cells compared to LPS and PGN exposure. Furthermore, PTX markedly decreased LPS-induced and, to a lesser degree, also PGN-induced intracellular IL-1β expression, but not FSL-1-induced intracellular IL-1β in cord blood monocytes (Fig. 7f).

## DISCUSSION

Newborn sepsis, in part mediated through the host inflammatory response to microbial invasion, is associated with high mortality and morbidity characterized by multiple organ damage, including perinatal brain injury (5, 6), for which the phosphodiesterase inhibitor PTX is a candidate adjunctive therapy (11, 26). Based on our previous findings on the inhibition of TLR-mediated proinflammatory cytokine production in newborn cord blood by PTX (13), improved bacterial clearance (20), and survival in septic newborn animals (27), we hypothesized that PTX decreases live bacterium-induced inflammatory cytokine production in newborn blood without enhancing bacterial proliferation *in vitro*. In order to mirror the pathological conditions of neonatal sepsis and to maximize the relevance of our *in vitro* studies, we studied for the first time the effects of PTX on inflammatory responses to clinically relevant live microorganisms that represent early and late onset sepsis pathogens (23) added to human newborn whole cord blood (21, 22, 28) and treated these samples with clinically relevant concentrations of PTX and antimicrobial agents (24, 25).

Our findings, which showed significantly higher induction of proinflammatory cytokine production by E. coli compared to Gram-positive bacteria, are consistent with previous reports (29, 30). PTX inhibited live microbe-induced proinflammatory cytokine transcription and production, with the most profound suppression directed toward TNF. In contrast, the transcription and production of the anti-inflammatory and proresolution cytokines IL-10 and IL-6 (31) were not decreased by PTX, thus favoring a polarization effect of PTX away from pro- and toward anti-inflammatory cytokine production. Similar findings have previously been reported for the inhibition of TLR agonist-induced proinflammatory cytokines in newborn and adult blood by PTX (13, 32, 33), which is consistent with its cAMP-dependent pathway (9). On the other hand, PTX under certain experimental conditions may also decrease the production of anti-inflammatory cytokines, including IL-10. PTX at high concentrations (1,000 µM) inhibited TNF-α, IL-1β, IL-6, CXCL-8, and IL-10 production in LPS-induced whole-blood cultures from healthy adult donors, whereas PTX at 100 µM only inhibited TNF-α (33). Moreover, PTX at 1,000 µM inhibited TNF-α, IL-1β, and IL-10 without changing IL-6 and CXCL-8 production in LPS-stimulated peripheral blood mononuclear cells, whereas IL-10 production was actually increased at 100 µM PTX (33). Although PTX inhibited TLR-mediated IL-10 production in newborn cord blood in our previous experiments, it did so to a lesser extent than toward TNF production, with a net effect of reducing the TNF/IL-10 ratio (13). While the effect of PTX on pro- and anti-inflammatory cytokine production may in part depend on drug concentrations and assay details (e.g., whole blood versus isolated peripheral blood mononuclear cells), there is an overall greater inhibitory effect of PTX on pro- versus anti-inflammatory cytokines. Our results on the inhibition of live microbe-induced proinflammatory cytokines in the presence of PTX were mirrored by comparable findings on PTX-mediated suppression of microbe-induced intracellular proinflammatory cytokines (TNF and IL-1β) and preservation of anti-inflammatory and proresolution cytokines in cord blood monocytes, as well as mRNA expression of cytokine genes in whole cord blood samples, thus further strengthening our experimental findings. Moreover, since the production of the inflammasome-mediated cytokine IL-1β is elevated in newborn compared to adult blood (34), the suppression of IL-1β in newborn cord blood and cord blood monocytes by PTX might be of particular relevance to protect the neonate from exaggerated inflammation in response to microbial invasion.

PTX in combination with antimicrobial agents, including GEN, VAN, and AMB, demonstrated significantly greater reduction of the live microbe-induced proinflammatory cytokines TNF and IL-1β than did PTX alone. Immunomodulatory effects have previously been described for macrolide antibiotics such as azithromycin (35, 36). Immunomodulatory effects, both pro- and anti-inflammatory, have also been reported for conventional antibiotics such as gentamicin and vancomycin, respectively (37–40). Concurrent administration of subtherapeutic doses of amphotericin B and a pentoxifylline analog led to increased survival times in experimental candidiasis in mice (41), whereas amphotericin B increased the expression of IL-1β in human mononuclear cells *in vitro* (42). However, findings on the immunological properties of conventional antimicrobial agents remain inconclusive to date, with variations in findings in part depending on the experimental design (43). We did not observe any effects on cytokine production when testing VAN, GEN, and AMB on TLR2 and TLR4 agonist-stimulated cord blood samples in the absence of live microorganisms. These observations suggest that antimicrobial-induced alterations of the tested live microorganisms E. coli, S. aureus, S. epidermidis, and C. albicans, including decreased viability (15), may have contributed to the measured reduction of proinflammatory cytokine production in our newborn blood samples. Taken together, these findings imply that maximal suppression of microbe-induced proinflammatory cytokine transcription and production by PTX may only be achievable in the context of appropriate antimicrobial therapy. This observation has potential clinical implications, i.e., it is possible that PTX in the context of suspected or confirmed infections, including sepsis, may be most helpful when used as an adjunctive agent in combination with appropriate antimicrobial therapy.

Whereas PTX alone or combined with GEN significantly suppressed E. coli-induced TNF and IL-1β expression in cord blood monocytes, PTX alone only led to a minor decrease in S. epidermidis-induced IL-1β without modifying intramonocytic TNF. Furthermore, PTX did not alter the expression of any S. epidermidis-induced cytokine-encoding genes, indicating a diminished capacity of PTX to inhibit S. epidermidis-induced inflammation. However, PTX led to diminished S. epidermidis-induced whole-blood supernatant TNF concentrations compared to untreated samples, which were further reduced in combination with VAN. Because of the apparent preferential anti-inflammatory effects of PTX toward E. coli-induced proinflammatory cytokines compared to the diminished or absent anti-inflammatory response toward S. epidermidis-induced inflammation in our neonatal blood samples, we examined the anti-inflammatory properties of PTX on TLR2 (PGN and FSL-1) findings versus TLR4 (LPS)-mediated cytokine production in whole cord blood and cord blood monocytes. These results indicated that the anti-inflammatory mechanisms of action of PTX may be independent of the TLR pathway engaged, as well as of the level of inflammation induced. On the other hand, PTX diminished FSL-1 (TLR2/6 agonist)-induced proinflammatory cytokine responses to a significantly lesser extent compared to PGN (TLR2 agonist, in addition to nucleotide oligomerization domain 1 and 2 signaling) and LPS, thus potentially exerting agonist-dependent differential immunomodulatory effects (44), an observation that warrants further investigation into the mechanisms of action involved.

In our experiments, PTX did not affect E. coli, S. epidermidis, S. aureus, and C. albicans CFU in newborn cord blood, suggesting that PTX does not enhance the proliferation and viability of these pathogens in newborn blood *in vitro*. However, these *in vitro* experimental findings should prompt future *in vivo* studies, including studies of neonatal sepsis animal models to investigate the effect of PTX on bacterial and fungal loads *in vivo*. Whereas PTX improved bacterial clearance in the context of hemorrhage and endotoxemia in rabbits (20), and decreased mortality from experimental S. aureus infection in newborn mice at lower doses (27), high-dose PTX actually increased mortality in S. aureus-infected newborn mice (27) and led to greater fungal burdens and shortened survival in cases of murine C. albicans sepsis (45). Based on limited clinical trials in human septic neonates, when administered as adjunct therapy to antibiotics, PTX may decrease mortality in cases of newborn sepsis (11). Studies employing live microbes in newborn animal sepsis models that examine microbial burden in blood and organ tissues, as well as morbidity and mortality, are needed to further characterize the concentration-dependent effects of PTX *in vivo*.

Our study contains several novel features. It is the first report to compare the anti-inflammatory effects of PTX toward inflammatory cytokine production induced by several live microorganisms that are most relevant to neonatal sepsis, including Gram-positive and Gram-negative bacteria, as well as fungal organisms. We further investigated the immunomodulatory properties of PTX in conjunction with the clinically relevant antimicrobial agents, and we monitored microbial proliferation under these experimental conditions. PTX, as well as all tested antimicrobial agents, was used at clinically relevant concentrations and tested in whole cord blood assays that contain all of the relevant cellular and humoral factors important for antimicrobial host immune responses (14, 46).

Along with these strengths, our study has also some limitations. While cord blood samples from healthy term newborns were used for our experiments, future studies should investigate the interactions of PTX, antimicrobial agents, and live microorganisms in blood samples obtained from clinically relevant target populations, including neonates exposed to perinatal infection and inflammation, as well as preterm newborns in whom innate immune responses and inflammatory cytokine production may not be fully developed at the time of birth (47). Intrapartum antibiotic therapy, e.g., in the context of chorioamnionitis, preterm premature membrane rupture, or Group B *Streptococcus* prophylaxis, resulting in potential active quantities of antibiotics in cord blood, may also change the responses to microbial stimulation of cord blood samples *in vitro* and should therefore be taken into account when studying immune responses of neonates exposed to intrauterine antimicrobial agents. Although we previously reported that PTX inhibited TLR-mediated proinflammatory cytokine production regardless of the timing of anti-inflammatory treatment in relation to TLR stimulation (13), the timing of administration of anti-inflammatory and antimicrobial agents in relation to the onset of sepsis may impact the efficacy and benefit of such treatment. Future studies should also consider investigating additional antimicrobial agents, including agents with intrinsic anti-inflammatory mechanisms of action such as azithromycin (35), and study the immunomodulatory effects of PTX on other Gram-positive and Gram-negative neonatal pathogens. Cord blood samples, which were used to model experimental newborn sepsis, may not be entirely predictive of inflammation-induced organ damage, since activated leukocytes in septic patients may lead to sustained inflammation in peripheral organs und subsequent organ damage (48). The *in vitro* findings on immunomodulatory effects of PTX in conjunction with antimicrobial agents should therefore be confirmed *in vivo* in a suitable newborn animal sepsis model such as the murine model.

In summary, our experimental findings demonstrated that PTX at clinically relevant concentrations, even as low as 10 μM, inhibited live microbe-induced proinflammatory cytokine production, especially when combined with clinically used antimicrobial agents, without increasing microbial colony counts in newborn cord blood, thus supporting its utility as candidate adjunctive anti-inflammatory agent for newborn sepsis.

## MATERIALS AND METHODS

### Subjects and human blood collection.

Collection of placental cord blood was approved by the Institutional Review Board of Stony Brook University, Stony Brook, NY, and written informed consent was obtained from study participants. Healthy term newborns of both sexes between 38 and 41 weeks gestation who delivered by Cesarean section without labor and without current infection, including HIV or documented intrauterine infection (i.e., absence of clinical chorioamnionitis, prolonged fetal membrane rupture over 12 h, clinical or laboratory signs of early-onset sepsis, or culture-proven sepsis of the mother or the newborn), were eligible (13). Cord blood was collected immediately after delivery of the placenta into sterile sodium heparin tubes containing 15 U/ml heparin (Becton, Dickinson, Franklin Lakes, NJ) through puncture of the veins on the fetal side of the placenta using sterile techniques, as previously described (13).

### Preparation of microorganisms.

Stimulation of cord blood samples was achieved with the live microorganisms E. coli K1 strain (ATCC 700973), S. epidermidis (ATCC 35984), S. aureus USA 300 strain 38 blood isolate (49), and C. albicans SC5314 clinical isolate (50). Single colonies of each microbial agent, stored on respective agar plates at 4°C, were grown overnight in their respective growth media (E. coli in Luria-Bertani [LB] broth, S. epidermidis and S. aureus in brain heart infusion (BHI) broth, and C. albicans in Sabouraud dextrose bouillon (SDA) broth, obtained from Becton Dickinson (Franklin Lakes, NJ), in a Forma Scientific orbital shaker (Thermo Fisher Scientific; Waltham, MA) at 150 rpm at 37°C to stationary phase. An aliquot of each microorganism was then transferred to fresh growth medium at 1:100 dilution and grown (150 rpm, 37°C) to exponential phase (E. coli for 2 h, S. epidermidis and S. aureus for 2.5 h, and C. albicans for 4 h). After centrifugation and washing of microbial suspensions in sterile saline, bacterial colonies per ml were determined spectrophotometrically at 600 nm and confirmed by plating of serial (1:10) dilutions and manually counting the CFU as described below. C. albicans concentration was determined using a hemocytometer. Microbial suspensions were diluted in sterile endotoxin-free saline to yield the desired inoculum concentration in cord blood samples. Antimicrobial (gentamicin, vancomycin, and amphotericin B) susceptibilities were confirmed by plating microorganisms onto agar plates containing different antimicrobial concentrations. The MIC was determined for each agent. Antimicrobial concentrations used in subsequent experiments were within clinically relevant concentration ranges and well above the MIC.

### *In vitro* stimulation and treatment of blood samples.

Cord blood samples were kept at room temperature and processed within 4 h of collection. Blood was diluted 1:1 with sterile prewarmed (37°C) RPMI 1640 (Life Technologies, Grand Island, NY), and a final volume of 200 μl was added to each well of 96-well tissue culture-treated round-bottom polystyrene plates (Becton Dickinson). Suspensions of live microorganisms were added to blood samples at 10 μl per well to yield the desired inoculum concentrations of 10^4^ CFU per ml of blood for E. coli and 10^5^ CFU per ml for S. epidermidis, S. aureus, and C. albicans. Blood samples were simultaneously treated with increasing molar concentrations of PTX (Tocris, Minneapolis, MN) and the respective antimicrobial agents, i.e., 12 µg/ml GEN (Life Technologies), 20 µg/ml VAN (Tocris), or 2 µg/ml AMB, combined PTX and antimicrobial agents, or vehicle control, and cultured at 37°C in a humidified incubator at 5% CO_2_ for the respective duration for each experimental protocol. Samples for bacterial plating were harvested after 0, 2, and 4 h in culture and serially (1:10) diluted using sterile endotoxin-free saline. Then, 100-µl aliquots each were plated onto LB (E. coli), BHI (S. epidermidis and S. aureus), or SDA (C. albicans) agar plates (Becton Dickinson), respectively. After incubation for 18 to 24 h (E. coli, S. epidermidis, and S. aureus) or 48 h (C. albicans) at 37°C, microbial colonies were manually counted with an eCount colony counter (Heathrow Scientific, Vernon Hills, IL). Upon culture completion, samples for supernatant cytokine measurements were centrifuged *in situ* at 500 × g for 10 min at room temperature, and approximately 120-μl portions of supernatants per well were carefully collected without disturbing the cell pellets, as previously described (13), and stored at –80°C until analyzed. For experiments involving TLR2 and TLR4 stimulation, purified PGN (peptidoglycan from S. aureus), FSL-1 (a synthetic diacylated lipoprotein and TLR2/6 agonist), and ultrapure LPS from E. coli O111:B4 were added to blood samples at the indicated concentrations (InvivoGen, San Diego, CA). Samples for mRNA expression and flow cytometry were processed as described below. Duplicate technical replicates were used for all immunoassays and flow cytometry experiments, whereas real-time PCR experiments were performed in triplicates. The number of independently conducted experiments utilizing blood samples from different donors is indicated for each experiment. The optimal duration of microbial stimulation for the different experimental procedures was determined through prior kinetic studies.

### Measurement of cytokine concentrations in culture supernatants.

Supernatant cytokine concentrations were determined with Bio-Plex Pro magnetic multiplex assays (Bio-Rad, Hercules, CA) and analyzed on the Bio-Plex 200 system with Bio-Plex Manager 5.0 software (Bio-Rad). Cytokine concentrations of samples stimulated with live microbes and treated with PTX and/or antimicrobial agents are expressed as a percentage compared to microbe-stimulated samples alone, which were defined as 100%.

### Real-time PCR.

Total RNA was isolated from cultured whole blood after erythrocyte lysis using QIAamp RNA blood minikits (Qiagen, Valencia, CA). Genomic DNA was removed with RNase-free DNase (Qiagen). The concentration and purity of RNA was measured with a NanoDrop ND-1000 spectrophotometer (NanoDrop Technologies, Wilmington, DE), as previously described (14), yielding ∼30 ng/µl RNA. Reverse transcription employed high-capacity cDNA reverse transcription kits (Life Technologies), and transcribed cDNA samples were used for real-time PCR-based mRNA expression measurements, which were performed in triplicates with 5 ng of cDNA per 10-μl reaction. Real-time PCR was performed on a StepOne Plus Real-time PCR system (Life Technologies) using TaqMan Fast Advanced Master Mix and TaqMan gene expression assays (Life Technologies). Beta actin served as normalization control and was multiplexed into all reaction wells. In order to ensure noninterference of the housekeeping gene with the target genes, all gene expression assays were first pretested by comparing their amplification efficiencies as a single-plex assay with their amplification efficiencies when multiplexed with the housekeeping gene (13). Data were analyzed using the ΔΔ threshold cycle (ΔΔ*C_T_*) method (51).

### Flow cytometry.

Cord blood samples were prepared with the Cytofix/Cytoperm fixation/permeabilization kit (BD Biosciences) with (TNF, IL-6, and IL-10) or without (IL-1β, TLR surface expression) the addition of brefeldin A, as previously described (14). Samples were stimulated with live E. coli (10^4^ CFU/ml blood) or S. epidermidis (10^5^ CFU/ml) and simultaneously treated with 200 μM PTX, antimicrobial agents (GEN or VAN, respectively), combined PTX and antibiotics, or vehicle control and then cultured at 37°C in 5% CO_2_ for 4 h. After surface staining, red blood cell lysis employed FACS lysing solution (BD). After fixation and permeabilization, samples were stained with monoclonal antibodies (PE-Cy7-conjugated mouse anti-TNF/clone MAb11, PE-conjugated mouse anti-IL-1β/clone AS10, PE-conjugated mouse anti-IL-6/clone AS12, APC-conjugated rat anti-IL-10/clone JES3-19F1, BV421-conjugated mouse-anti-human CD282/clone 11G7, and PE-conjugated mouse anti-CD484/clone TF901 [all from BD]) or their corresponding isotype controls. Samples were analyzed on an LSR Fortessa flow cytometer (BD). Compensation beads (BD) were used as single-stain positive and negative controls. Monocytes were gated with forward and side scatter as CD45^+^ CD14^+^ cells (PerCP-Cy5.5-conjugated mouse anti-CD45/clone 2D1, FITC-conjugated mouse anti-CD14/clone M5E2, or APC-conjugated mouse anti-CD14/clone MϕP9; BD). Data from 10,000 monocytes were acquired for each condition and analyzed using Kaluza version 1.3 software (Beckman Coulter, Jersey City, NJ). The geometric mean fluorescence intensity (MFI) of all monocytes was determined after subtraction of isotype controls.

Samples for measurements of MAPK phosphorylation and total IκBα were cultured for 30 min, followed by immediate red blood cell lysis and fixation using Lyse/Fix buffer (BD). After permeabilization with Perm Buffer II (BD), samples were simultaneously stained with the appropriate surface and intracellular antibodies (AF647-conjugated mouse anti-p38[pT180/pY182]/clone 36 and AF647-conjugated mouse anti-IĸBα/clone 25/IĸBα/MAD-3).

### Caspase 1 activation.

Cord blood samples were stimulated with live E. coli or S. epidermidis and simultaneously treated with 200 μM PTX, antibiotics (GEN or VAN), combined PTX and antibiotics, or vehicle control. Samples were cultured for 1 h at 37°C in 5% CO_2_ in the presence of Fluorescent Labeled Inhibitor of Caspases (FLICA) reagent (ImmunoChemistry Technologies, LLC, Bloomington, MN) and stained for monocyte surface markers, as previously described (14). Upon completion of cultures, samples were immediately subjected to red blood cell lysis and fixation using Lyse/Fix buffer (BD), washed, and analyzed on an LSR Fortessa flow cytometer (BD). MFI data from 10,000 CD45^+^ CD14^+^ monocytes, gated on forward and side scatter, were acquired for each condition as described above and analyzed using Kaluza version 1.3 software (Beckman Coulter). The geometric MFI of all monocytes was determined after subtraction of background fluorescence (no FLICA reagent).

### Statistical analysis.

Supernatant cytokine concentrations of samples stimulated with live microorganisms and treated with PTX and/or antimicrobial agents were expressed as a percentage compared to microbe-stimulated samples alone, which were defined as 100%. Linear mixed models, which take into account the possible dependence among measurements from the same sample under different treatment conditions, were used to analyze drug concentration response data for each cytokine, microorganism, and treatment condition independently. The covariance structure among drug concentrations within a subject was modeled as compound symmetry.

Flow cytometric MFI data were presented as fold changes normalized to microbial stimulation alone. Linear mixed models were employed to analyze each combination of stimulation and target analyte independently. The covariance structure among different treatment conditions within the same subject was modeled as compound symmetry.

Gene expression data were analyzed by comparing ΔΔ*C_T_* values for each gene and microorganism independently. Gene expression under different treatment conditions (i.e., antimicrobial and/or anti-inflammatory agents) were evaluated as differences compared to the control condition (microbial stimulation without treatment). For repeated measure data, linear mixed models were performed to control the within-subject dependence under different treatments for the same subject. To model the within-subject dependence robustly, the covariance matrices among different treatments were modeled as unstructured variances. Means and standard deviations were estimated and compared between treatments employing F tests based on the linear mixed models. For graphic presentation only, relative mRNA expression values of samples stimulated with microorganisms and treated with PTX and/or antimicrobial agents were calculated from ΔΔ*C_T_* values and normalized to microbial stimulation alone, which was defined as 100%. SAS 9.3 statistical software (SAS Institute, Inc., Cary, NC) was used for analyses, and Prism v6.01 (GraphPad Software, San Diego, CA) was used to graph the results. All statistical tests were two sided, and *P* values were adjusted for multiple testing using a false discovery rate (*P* < 0.05).

## Supplementary Material

Supplemental file 1

## References

[1] Lawn JE, Cousens S, Zupan J 2005 4 million neonatal deaths: When? Where? Why? Lancet 365:891–900. doi:10.1016/S0140-6736(05)71048-5. 15752534

[2] Goldenberg RL, Hauth JC, Andrews WW 2000 Intrauterine infection and preterm delivery. N Engl J Med 342:1500–1507. doi:10.1056/NEJM200005183422007. 10816189

[3] Redondo AC, Ceccon ME, Silveira-Lessa AL, Quinello C, Palmeira P, Carvalho WB, Carneiro-Sampaio M 2014 TLR-2 and TLR-4 expression in monocytes of newborns with late-onset sepsis. J Pediatr (Rio J) 90:472–478. doi:10.1016/j.jped.2013.12.012. 24878008

[4] Bi D, Qiao L, Bergelson I, Ek CJ, Duan L, Zhang X, Albertsson AM, Pettengill M, Kronforst K, Ninkovic J, Goldmann D, Janzon A, Hagberg H, Wang X, Mallard C, Levy O 2015 *Staphylococcus epidermidis* bacteremia induces brain injury in neonatal mice via Toll-like receptor 2-dependent and -independent pathways. J Infect Dis 212:1480–1490. doi:10.1093/infdis/jiv231. 25883383 PMC4601917

[5] Strunk T, Inder T, Wang X, Burgner D, Mallard C, Levy O 2014 Infection-induced inflammation and cerebral injury in preterm infants. Lancet Infect Dis 14:751–762. doi:10.1016/S1473-3099(14)70710-8. 24877996 PMC4125363

[6] Wynn J, Cornell TT, Wong HR, Shanley TP, Wheeler DS 2010 The host response to sepsis and developmental impact. Pediatrics 125:1031–1041. doi:10.1542/peds.2009-3301. 20421258 PMC2894560

[7] Cornette L 2004 Fetal and neonatal inflammatory response and adverse outcome. Semin Fetal Neonatal Med 9:459–470. doi:10.1016/j.siny.2004.08.004. 15691784

[8] Du Pont-Thibodeau G, Joyal JS, Lacroix J 2014 Management of neonatal sepsis in term newborns. F1000Prime Rep 6:67. doi:10.12703/P6-67. 25165566 PMC4126544

[9] Serezani CH, Ballinger MN, Aronoff DM, Peters-Golden M 2008 Cyclic AMP: master regulator of innate immune cell function. Am J Respir Cell Mol Biol 39:127–132. doi:10.1165/rcmb.2008-0091TR. 18323530 PMC2720142

[10] Minguet S, Huber M, Rosenkranz L, Schamel WW, Reth M, Brummer T 2005 Adenosine and cAMP are potent inhibitors of the NF-κB pathway downstream of immunoreceptors. Eur J Immunol 35:31–41. doi:10.1002/eji.200425524. 15580656

[11] Pammi M, Haque KN 2015 Pentoxifylline for treatment of sepsis and necrotizing enterocolitis in neonates. Cochrane Database Syst Rev CD004205. 10.1002/14651858.CD004205.pub2 21975745

[12] Australian New Zealand Clinical Trials Registry. 2018 Can pentoxifylline improve long-term outcomes in preterm infants with late-onset sepsis or necrotizing enterocolitis? A pragmatic, randomized, placebo-controlled trial. Australian New Zealand Clinical Trials Registry ACTRN12616000405415 https://www.anzctr.org.au/Trial/Registration/TrialReview.aspx?id=370404.

[13] Speer EM, Dowling DJ, Ozog LS, Xu J, Yang J, Kennady G, Levy O 2017 Pentoxifylline inhibits TLR- and inflammasome-mediated *in vitro* inflammatory cytokine production in human blood with greater efficacy and potency in newborns. Pediatr Res 81:806–816. doi:10.1038/pr.2017.6. 28072760

[14] Speer EM, Dowling DJ, Xu J, Ozog LS, Mathew JA, Chander A, Yin D, Levy O 2018 Pentoxifylline, dexamethasone and azithromycin demonstrate distinct age-dependent and synergistic inhibition of TLR- and inflammasome-mediated cytokine production in human newborn and adult blood *in vitro*. PLoS One 13:e0196352. doi:10.1371/journal.pone.0196352. 29715306 PMC5929513

[15] Sander LE, Davis MJ, Boekschoten MV, Amsen D, Dascher CC, Ryffel B, Swanson JA, Müller M, Blander JM 2011 Detection of prokaryotic mRNA signifies microbial viability and promotes immunity. Nature 474:385–389. doi:10.1038/nature10072. 21602824 PMC3289942

[16] Mourao-Sa D, Roy S, Blander MJ 2013 Vita-PAMPs: signatures of microbial viability. Adv Exp Med Biol 785:1–8. doi:10.1007/978-1-4614-6217-0_1. 23456832

[17] Strunk T, Richmond P, Prosser A, Simmer K, Levy O, Burgner D, Currie A 2011 Method of bacterial killing differentially affects the human innate immune response to *Staphylococcus epidermidis*. Innate Immun 17:508–516. doi:10.1177/1753425910379840. 20807722

[18] Rettig L, Haen SP, Bittermann AG, von Boehmer L, Curioni A, Krämer SD, Knuth A, Pascolo S 2010 Particle size and activation threshold: a new dimension of danger signaling. Blood 115:4533–4541. doi:10.1182/blood-2009-11-247817. 20304804

[19] Ugolini M, Gerhard J, Burkert S, Jensen KJ, Georg P, Ebner F, Volkers SM, Thada S, Dietert K, Bauer L, Schäfer A, Helbig ET, Opitz B, Kurth F, Sur S, Dittrich N, Gaddam S, Conrad ML, Benn CS, Blohm U, Gruber AD, Hutloff A, Hartmann S, Boekschoten MV, Müller M, Jungersen G, Schumann RR, Suttorp N, Sander LE 2018 Recognition of microbial viability via TLR8 drives T(FH) cell differentiation and vaccine responses. Nat Immunol 19:386–396. doi:10.1038/s41590-018-0068-4. 29556002

[20] Heller S, Weber K, Heller A, Urbaschek R, Koch T 1999 Pentoxifylline improves bacterial clearance during hemorrhage and endotoxemia. Crit Care Med 27:756–763. doi:10.1097/00003246-199904000-00031. 10321666

[21] Belderbos ME, Levy O, Meyaard L, Bont L 2013 Plasma-mediated immune suppression: a neonatal perspective. Pediatr Allergy Immunol 24:102–113. doi:10.1111/pai.12023. 23173652

[22] Elahi S, Ertelt JM, Kinder JM, Jiang TT, Zhang X, Xin L, Chaturvedi V, Strong BS, Qualls JE, Steinbrecher KA, Kalfa TA, Shaaban AF, Way SS 2013 Immunosuppressive CD71^+^ erythroid cells compromise neonatal host defence against infection. Nature 504:158–162. doi:10.1038/nature12675. 24196717 PMC3979598

[23] Cortese F, Scicchitano P, Gesualdo M, Filaninno A, De Giorgi E, Schettini F, Laforgia N, Ciccone MM 2016 Early and late infections in newborns: Where do we stand? A review. Pediatr Neonatol 57:265–273. doi:10.1016/j.pedneo.2015.09.007. 26750406

[24] Szymura-Oleksiak J, Bury J, Lauterbach R, Pawlowski M 1997 Serum concentrations of pentoxifylline and its metabolites in premature infants with sepsis when administered by continuous intravenous infusion. Pharm Sci 3:367–371.

[25] Page-Sharp M, Strunk T, Salman S, Hibbert J, Patole SK, Manning L, Batty KT 2017 Simultaneous determination of pentoxifylline, metabolites M1 (lisofylline), M4 and M5, and caffeine in plasma and dried blood spots for pharmacokinetic studies in preterm infants and neonates. J Pharm Biomed Anal 46:302–313. doi:10.1016/j.jpba.2017.08.030. 28903089

[26] Tarnow-Mordi W, Isaacs D, Dutta S 2010 Adjunctive immunologic interventions in neonatal sepsis. Clin Perinatol 37:481–499. doi:10.1016/j.clp.2009.12.002. 20569818

[27] Maderazo EG, Breaux S, Woronick CL, Krause PJ 1990 Efficacy, toxicity, and pharmacokinetics of pentoxifylline and its analogs in experimental *Staphylococcus aureus* infections. Antimicrob Agents Chemother 34:1100–1106. doi:10.1128/AAC.34.6.1100. 2393269 PMC171765

[28] Levy O, Coughlin M, Cronstein BN, Roy RM, Desai A, Wessels MR 2006 The adenosine system selectively inhibits TLR-mediated TNF-α production in the human newborn. J Immunol 177:1956–1966. doi:10.4049/jimmunol.177.3.1956. 16849509 PMC2881468

[29] Ogata M, Nandate K, Kawasaki T, Kawasaki C, Ozaki M, Shigematsu A 2004 A platelet activating factor receptor antagonist inhibits cytokine production in human whole blood by bacterial toxins and live bacteria. Anesth Analg 98:1767–1772. 15155343 10.1213/01.ANE.0000112310.93297.AF

[30] Frieling JT, Mulder JA, Hendriks T, Curfs JH, van der Linden CJ, Sauerwein RW 1997 Differential induction of pro- and anti-inflammatory cytokines in whole blood by bacteria: effects of antibiotic treatment. Antimicrob Agents Chemother 41:1439–1443. 9210662 10.1128/aac.41.7.1439 PMC163936

[31] Jones SA 2005 Directing transition from innate to acquired immunity: defining a role for IL-6. J Immunol 175:3463–3468. doi:10.4049/jimmunol.175.6.3463. 16148087

[32] Schüller SS, Wisgrill L, Herndl E, Spittler A, Förster-Waldl E, Sadeghi K, Kramer BW, Berger A 2017 Pentoxifylline modulates LPS-induced hyperinflammation in monocytes of preterm infants *in vitro*. Pediatr Res 82:215–225. doi:10.1038/pr.2017.41. 28288151

[33] D’Hellencourt CL, Diaw L, Cornillet P, Guenounou M 1996 Differential regulation of TNF-α, IL-1β, IL-6, IL-8, TNF-β, and IL-10 by pentoxifylline. Int J Immunopharmacol 18:739–748. doi:10.1016/S0192-0561(97)85556-7. 9172017

[34] Dowling DJ, Levy O 2014 Ontogeny of early life immunity. Trends Immunol 35:299–310. doi:10.1016/j.it.2014.04.007. 24880460 PMC4109609

[35] Aghai ZH, Kode A, Saslow JG, Nakhla T, Farhath S, Stahl GE, Eydelman R, Strande L, Leone P, Rahman I 2007 Azithromycin suppresses activation of nuclear factor-κB and synthesis of proinflammatory cytokines in tracheal aspirate cells from premature infants. Pediatr Res 62:483–488. doi:10.1203/PDR.0b013e318142582d. 17667842

[36] Vrančić M, Banjanac M, Nujić K, Bosnar M, Murati T, Munić V, Stupin Polančec D, Belamarić D, Parnham MJ, Eraković Haber V 2012 Azithromycin distinctively modulates classical activation of human monocytes *in vitro*. Br J Pharmacol 165:1348–1360. doi:10.1111/j.1476-5381.2011.01576.x. 21726210 PMC3372721

[37] Zager RA, Johnson AC, Geballe A 2007 Gentamicin suppresses endotoxin-driven TNF-alpha production in human and mouse proximal tubule cells. Am J Physiol Renal Physiol 293:F1373–F1380. doi:10.1152/ajprenal.00333.2007. 17699551

[38] Bode C, Diedrich B, Muenster S, Hentschel V, Weisheit C, Rommelsheim K, Hoeft A, Meyer R, Boehm O, Knuefermann P, Baumgarten G 2014 Antibiotics regulate the immune response in both presence and absence of lipopolysaccharide through modulation of Toll-like receptors, cytokine production and phagocytosis *in vitro*. Int Immunopharmacol 18:27–34. doi:10.1016/j.intimp.2013.10.025. 24239744

[39] Bode C, Muenster S, Diedrich B, Jahnert S, Weisheit C, Steinhagen F, Boehm O, Hoeft A, Meyer R, Baumgarten G 2015 Linezolid, vancomycin and daptomycin modulate cytokine production, Toll-like receptors and phagocytosis in a human *in vitro* model of sepsis. J Antibiot (Tokyo) 68:485–490. doi:10.1038/ja.2015.18. 25735844 PMC4579589

[40] Al-Banna NA, Pavlovic D, Gründling M, Zhou J, Kelly M, Whynot S, Hung O, Johnston B, Issekutz TB, Kern H, Cerny V, Lehmann C 2013 Impact of antibiotics on the microcirculation in local and systemic inflammation. Clin Hemorheol Microcirc 53:155–169. doi:10.3233/CH-2012-1583. 22975936

[41] Wasan KM, Vadiei K, Luke DR, Keyhani A, White RA, McQueen TJ, Mehta R, Lopez-Berestein G 1991 Antifungal activity of HWA-138 and amphotericin B in experimental systemic candidiasis. Antimicrob Agents Chemother 35:2046–2048. doi:10.1128/AAC.35.10.2046. 1759826 PMC245323

[42] Cleary JD, Chapman SW, Nolan RL 1992 Pharmacologic modulation of interleukin-1 expression by amphotericin B-stimulated human mononuclear cells. Antimicrob Agents Chemother 36:977–981. doi:10.1128/AAC.36.5.977. 1510423 PMC188802

[43] Krehmeier U, Bardenheuer M, Voggenreiter G, Obertacke U, Schade FU, Majetschak M 2002 Effects of antimicrobial agents on spontaneous and endotoxin-induced cytokine release of human peripheral blood mononuclear cells. J Infect Chemother 8:194–197. doi:10.1007/s101560200036. 12111578

[44] Wetzler LM 2003 The role of Toll-like receptor 2 in microbial disease and immunity. Vaccine 21(Suppl 2):S55–S60. 12763684 10.1016/s0264-410x(03)00201-9

[45] Louie A, Baltch AL, Franke MA, Ritz WJ, Smith RP, Singh JK, Gordon MA 1996 Effect of pentoxifylline on the course of systemic *Candida albicans* infection in mice. J Antimicrob Chemother 37:943–954. doi:10.1093/jac/37.5.943. 8737144

[46] Dowling DJ, van Haren SD, Scheid A, Bergelson I, Kim D, Mancuso CJ, Foppen W, Ozonoff A, Fresh L, Theriot TB, Lackner AA, Fichorova RN, Smirnov D, Vasilakos JP, Beaurline JM, Tomai MA, Midkiff CC, Alvarez X, Blanchard JL, Gilbert MH, Aye PP, Levy O 2017 TLR7/8 adjuvant overcomes newborn hyporesponsiveness to pneumococcal conjugate vaccine at birth. JCI Insight 2:e91020. doi:10.1172/jci.insight.91020. 28352660 PMC5360187

[47] Strunk T, Prosser A, Levy O, Philbin V, Simmer K, Doherty D, Charles A, Richmond P, Burgner D, Currie A 2012 Responsiveness of human monocytes to the commensal bacterium *Staphylococcus epidermidis* develops late in gestation. Pediatr Res 72:10–18. doi:10.1038/pr.2012.48. 22460219

[48] Cavaillon JM, Eisen D, Annane D 2014 Is boosting the immune system in sepsis appropriate?. Crit Care 18:216. doi:10.1186/cc13787. 24886820 PMC4035855

[49] Varshney AK, Mediavilla JR, Robiou N, Guh A, Wang X, Gialanella P, Levi MH, Kreiswirth BN, Fries BC 2009 Diverse enterotoxin gene profiles among clonal complexes of *Staphylococcus aureus* isolates from the Bronx, New York. Appl Environ Microbiol 75:6839–6849. doi:10.1128/AEM.00272-09. 19749060 PMC2772442

[50] Gillum AM, Tsay EY, Kirsch DR 1984 Isolation of the *Candida albicans* gene for orotidine-5′-phosphate decarboxylase by complementation of *S. cerevisiae ura3* and *E. coli pyrF* mutations. Mol Gen Genet 198:179–182. doi:10.1007/BF00328721. 6394964

[51] Pestana EA, Belak S, Diallo A, Crowther JR, Viljoen GJ 2010 Early, rapid, and sensitive veterinary molecular diagnostics: real time PCR applications, p 247–263. Springer Science and Business Media, Dordrecht, Netherlands.

