# Supplementary material for: Pentoxifylline Alone or in Combination with Gentamicin or Vancomycin Inhibits Live Microbe-Induced Proinflammatory Cytokine Production in Human Cord Blood and Cord Blood Monocytes In Vitro
Source: Antimicrob Agents Chemother. 2018 Nov 26;62(12):e01462-18. doi: 10.1128/AAC.01462-18 (PMC6256750; doi:10.1128/AAC.01462-18)
Supplement: Supplemental file 1 [file zac012187661s1.pdf]

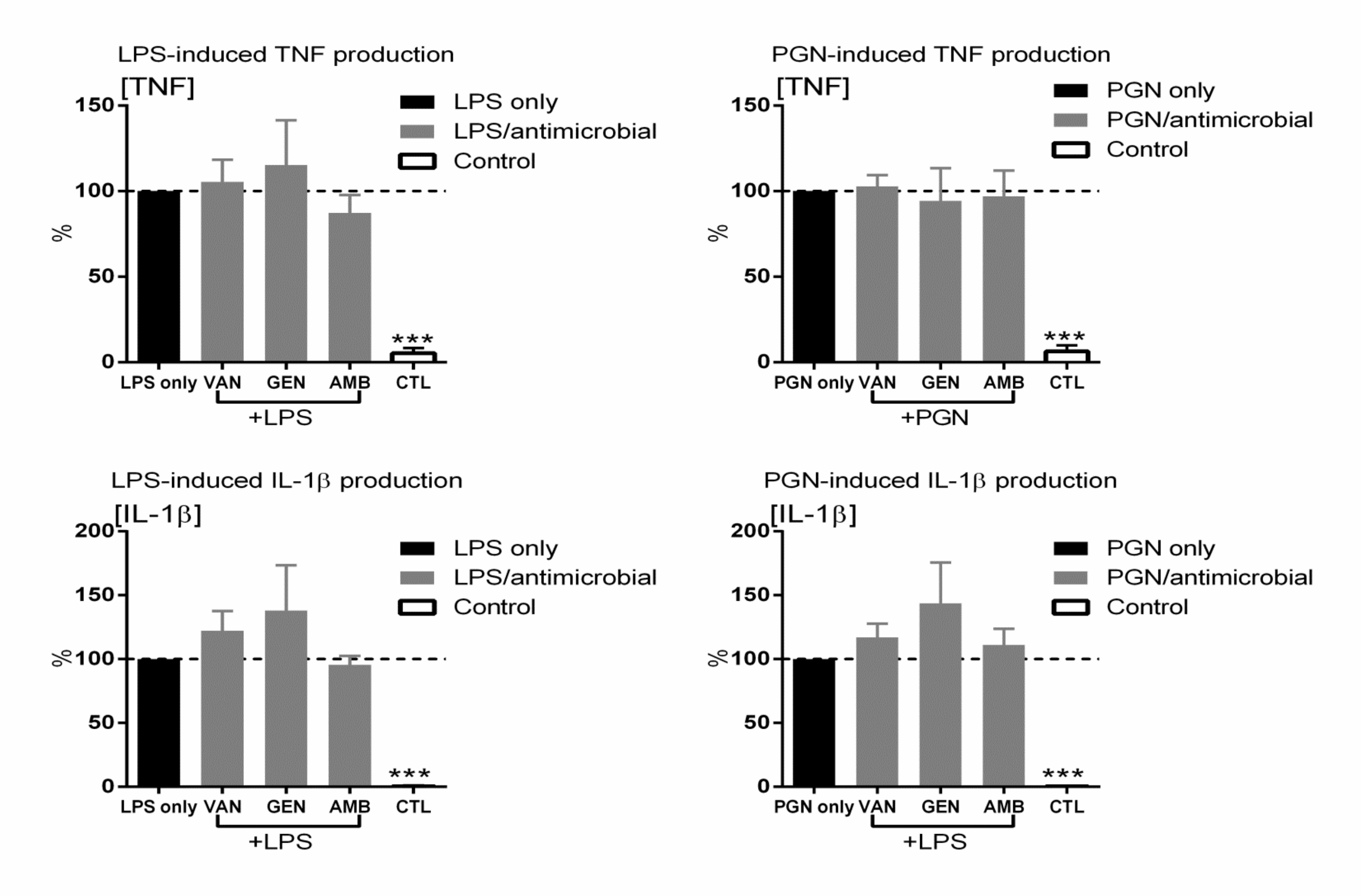

FIG S1: No intrinsic anti-inflammatory effects of VAN, GEN and AMB on TLR agonist-induced cytokine production in human cord blood. Whole cord blood (n=5) was stimulated with TLR2 (PGN 10 µg/ml) or TLR4 (LPS 10 ng/ml) agonists and simultaneously treated with VAN, GEN, AMB, or vehicle control. Samples were cultured for 6 hours in 5% CO_2 at 37º C. Samples treated with antimicrobial agents were expressed as a percentage compared to samples stimulated with TLR agonists only (reference samples), which were defined as 100%. Significant differences of treated samples compared to the corresponding reference samples were indicated: *p<0.05, **p<0.01, ***p<0.001.

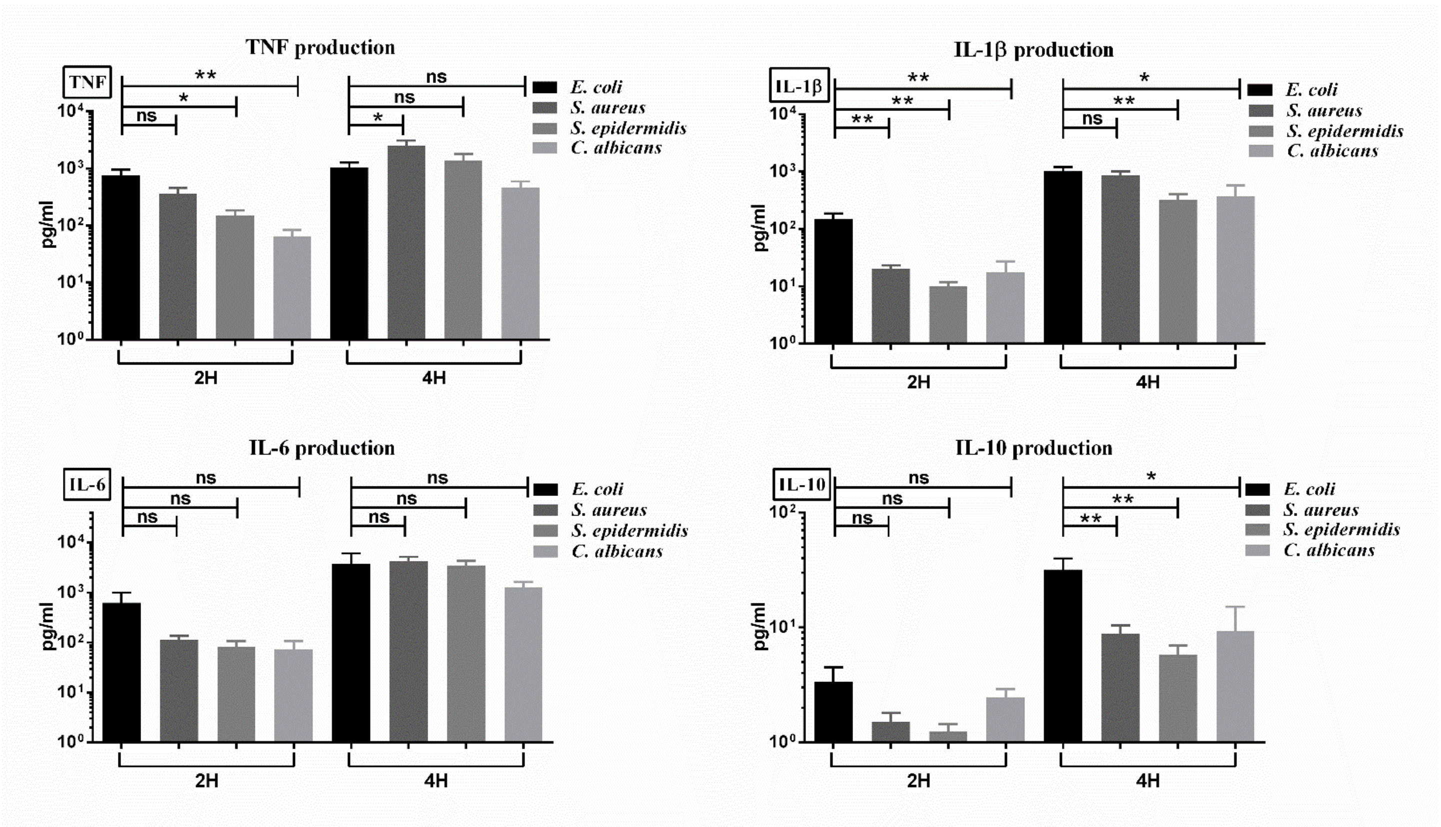

FIG S2: Microbe-induced cytokine production in term human cord blood. Whole cord blood (n=10 to 13) was stimulated with live microorganisms: 10^4 CFU/ml *E. coli*, 10^5 CFU/ml *S. aureus*, 10^5 CFU/ml *S. epidermidis,* and 10^5 CFU/ml *C. albicans,* and cultured for 2 or 4 hours in 5% CO_2 at 37°C. Supernatant cytokines were measured with multiplex immunoassays, and comparisons of cytokine concentrations from *E. coli*-stimulated vs *S. aureus*-, *S. epidermidis*- or *C. albicans*–stimulated blood samples are shown. Significant differences based on paired t tests were indicated: *p<0.05, **p<0.01, ***p<0.001.

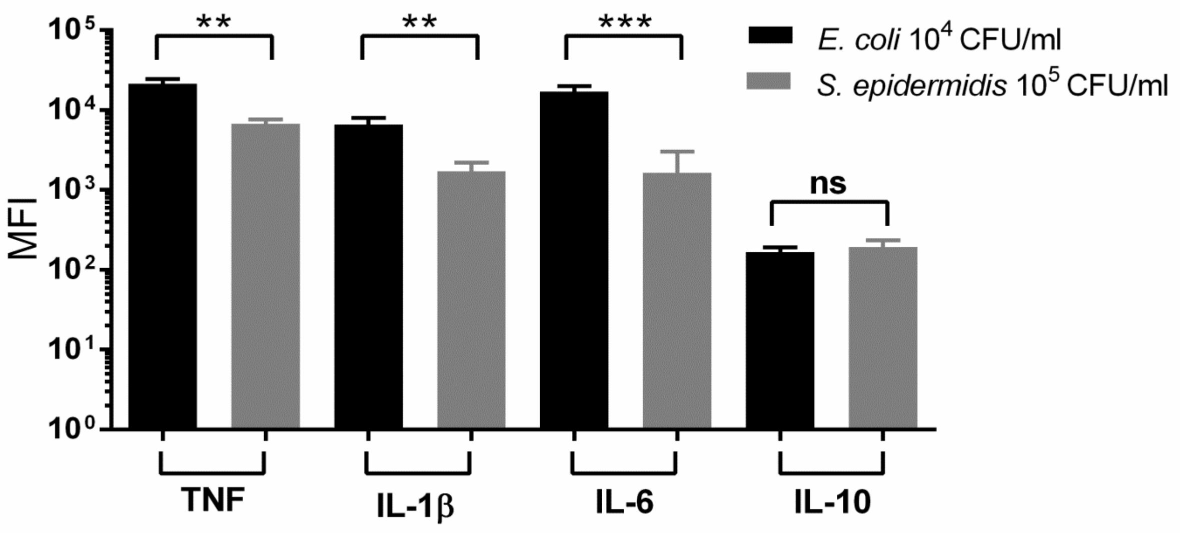

FIG S3: Microbe-induced intracellular cytokine staining in CD14$^+$CD45$^+$ cord blood monocytes. Whole cord blood (n=10) was stimulated with live *E. coli* or *S. epidermidis,* and cultured for 4 hours in 5% CO_2 at 37°C. Samples were subjected to intracellular cytokine-staining for flow cytometry. Comparisons of MFI values from *E. coli*-stimulated vs *S. epidermidis*–stimulated blood samples are shown. Significant differences based on paired t tests were indicated: *$p<0.05$, **$p<0.01$, ***$p<0.001$.

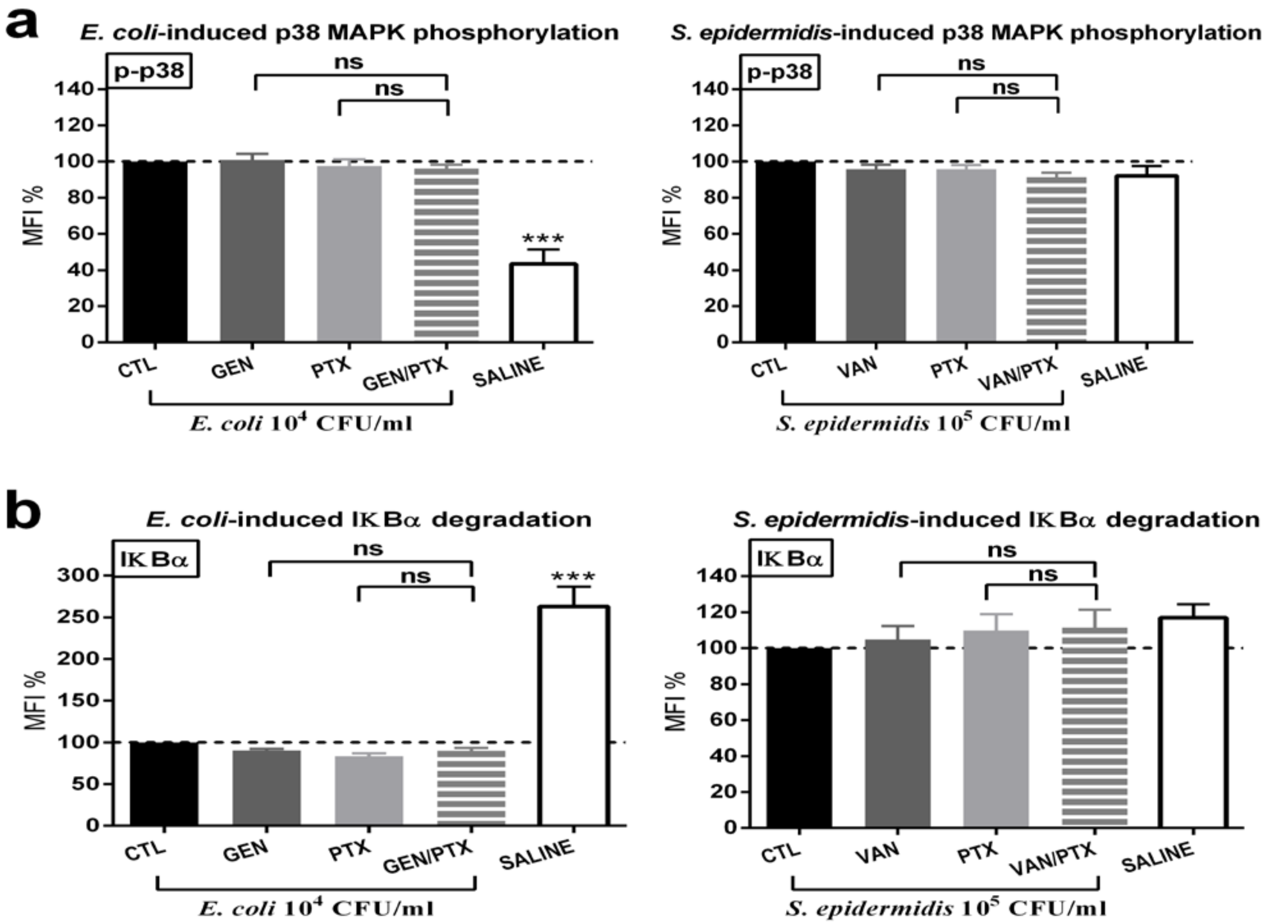

FIG S4: Lack of inhibition of *E. coli*- and *S. epidermidis*-induced MAPK p38 phosphorylation and IκBα degradation in cord blood monocytes in the presence of PTX and/or antibiotics. Whole cord blood (n=10) was stimulated with live *E. coli* or *S. epidermidis*, treated with 200 μM PTX and/or antibiotics or vehicle control, and cultured for 30 min in 5% CO_2 at 37°C. Samples were subjected to intracellular staining for a) phosphorylated p38 MAPK and b) total IκBα for flow cytometric MFI measurements. Comparisons of MFI values from *E. coli*-stimulated vs *S. epidermidis*–stimulated blood samples are shown. Significant differences between treated compared to untreated samples and between samples treated with single (antimicrobial or anti-inflammatory) agents compared to combination treatment based on linear mixed model t tests, adjusted for multiple comparisons employing false discovery rates, were indicated: *p<0.05, **p<0.01, ***p<0.001.

Table S1: PTX and antimicrobial agents alone or combined inhibited mRNA gene expression of pro-inflammatory cytokines in newborn cord blood

Gene	Microorganism	Culture duration	Mean ΔΔCT (compared to microbial stimulation alone)		
			PTX[a]	ANTIMICRO-BIAL[a]	PTX + ANTI-MICROBIAL[a]
TNF	*E. coli*	1H	↓↓ 1.58	Ø 0.40	↓↓ 1.92
		2H	↓ 2.12	↓ 1.59	↓↓ 2.34
	S. aureus	1H	↓ 1.20	Ø -0.16	↓ 0.82
		2H	↓ 0.83	↓↓ 1.18	↓↓↓ 2.36
	S. epidermidis	1H	Ø 0.84	Ø 0.42	Ø 0.95
		2H	Ø 1.11	↓ 1.57	↓ 1.93
	C. albicans	1H	↓ 0.74	Ø -0.63	Ø 0.82
		2H	↓↓ 0.98	Ø -0.36	↓ 0.86
IL1B	*E. coli*	1H	Ø 0.56	Ø 0.47	Ø 0.89
		2H	Ø 0.82	↓ 1.20	↓ 0.87
	S. aureus	1H	Ø 0.75	Ø -0.38	Ø 0.15
		2H	Ø 0.24	↓↓ 0.90	↓↓ 1.31
	S. epidermidis	1H	Ø 0.36	Ø 0.82	Ø 0.83
		2H	Ø 0.64	↓ 1.80	↓ 1.50
	C. albicans	1H	↓ 0.77	Ø -0.33	Ø 0.54
		2H	Ø 0.61	Ø 0.04	Ø 0.40
IL6	*E. coli*	1H	Ø 0.48	Ø 0.74	Ø 0.75
		2H	Ø 1.01	↓ 1.80	↓ 1.41
	S. aureus	1H	Ø -0.63	Ø -0.91	↑ -1.32
		2H	Ø -0.36	↓↓ 1.13	↓ 1.08
	S. epidermidis	1H	Ø -0.81	Ø 0.64	Ø -0.83
		2H	Ø 0.13	↓↓ 2.98	Ø 1.85
	C. albicans	1H	↑↑ -1.12	↑ -1.70	↑ -1.91
		2H	Ø -0.41	Ø -1.06	↑ -1.64
IL10	*E. coli*	1H	Ø -0.23	Ø 0.17	Ø 0.07
		2H	Ø 0.98	Ø 1.22	Ø 0.82
	S. aureus	1H	Ø 0.50	Ø -0.14	Ø 0.31
		2H	Ø -0.57	Ø -0.59	Ø -0.26
	S. epidermidis	1H	Ø 0.13	Ø -0.15	Ø -0.21
		2H	Ø 0.20	Ø 0.13	Ø -0.31
	C. albicans	1H	Ø -0.52	Ø -0.30	Ø -0.05
		2H	Ø -0.33	↑ -0.65	Ø -0.59
TLR2	*E. coli*	1H	Ø 0.40	Ø 0.30	Ø 0.44
		2H	↓ 1.12	Ø 0.66	Ø 0.71
	S. aureus	1H	Ø 0.25	Ø -0.07	Ø 0.11
		2H	Ø 0.60	Ø 0.14	Ø 0.60
	S. epidermidis	1H	Ø 0.16	Ø 0.18	Ø -0.33
		2H	Ø 0.41	Ø 0.41	Ø 0.54
	C. albicans	1H	Ø -0.02	Ø -0.13	Ø 0.06

		2H	Ø	0.51	Ø	-0.08	Ø	0.41
TLR4	*E. coli*	1H	Ø	0.45	Ø	-0.38	Ø	0.03
		2H	Ø	0.45	Ø	-0.38	Ø	0.03
	S. aureus	1H	Ø	0.30	Ø	-0.14	Ø	0.32
		2H	Ø	0.30	Ø	-0.14	Ø	0.32
	S. epidermidis	1H	Ø	0.00	Ø	-0.32	Ø	-0.94
		2H	Ø	0.00	Ø	-0.32	Ø	-0.94
	C. albicans	1H	Ø	0.05	Ø	-0.79	Ø	0.44
		2H	Ø	0.05	Ø	-0.79	Ø	0.44
RELA	*E. coli*	1H	Ø	0.12	Ø	-0.43	Ø	0.03
		2H	Ø	0.90	Ø	1.10	Ø	0.63
	S. aureus	1H	Ø	-0.04	Ø	-0.19	Ø	-0.14
		2H	Ø	0.03	Ø	0.06	Ø	0.17
	S. epidermidis	1H	Ø	-0.26	Ø	-0.36	Ø	-0.78
		2H	Ø	-0.17	Ø	-0.09	Ø	-0.53
	C. albicans	1H	Ø	-0.46	Ø	-0.65	Ø	0.01
		2H	Ø	-0.41	Ø	-0.13	Ø	-0.48
NFKBIA	*E. coli*	1H	Ø	0.06	Ø	-0.20	Ø	0.10
		2H	Ø	1.08	Ø	1.07	Ø	1.03
	S. aureus	1H	Ø	0.03	Ø	-0.45	Ø	-0.13
		2H	Ø	0.51	↓	0.50	↓	1.04
	S. epidermidis	1H	Ø	0.08	Ø	0.18	Ø	-0.24
		2H	Ø	0.33	Ø	0.83	Ø	0.81
	C. albicans	1H	Ø	-1.01	Ø	-0.42	Ø	-0.25
		2H	Ø	0.00	Ø	0.21	Ø	0.28
DUSP1	*E. coli*	1H	Ø	-0.07	Ø	0.10	Ø	0.15
		2H	Ø	0.18	Ø	0.97	Ø	0.28
	S. aureus	1H	Ø	-0.36	Ø	-0.26	Ø	-0.51
		2H	Ø	-0.03	↓	0.83	Ø	0.63
	S. epidermidis	1H	Ø	-0.29	Ø	0.51	Ø	-0.26
		2H	Ø	-0.21	Ø	0.53	Ø	0.00
	C. albicans	1H	Ø	-0.04	Ø	0.42	Ø	0.23
		2H	Ø	-0.10	↓	0.67	Ø	0.20
CASP1	*E. coli*	1H	Ø	0.40	Ø	0.17	Ø	0.43
		2H	Ø	0.85	Ø	0.85	Ø	0.59
	S. aureus	1H	Ø	0.11	Ø	-0.38	Ø	0.09
		2H	Ø	0.21	Ø	-0.05	Ø	0.22
	S. epidermidis	1H	Ø	0.35	Ø	0.43	Ø	0.12
		2H	Ø	0.09	Ø	0.08	Ø	-0.06
	C. albicans	1H	Ø	0.42	Ø	0.25	Ø	0.47
		2H	Ø	0.17	Ø	-0.14	Ø	-0.10
NLRP3	*E. coli*	1H	Ø	0.10	Ø	0.35	Ø	0.26
		2H	Ø	0.30	Ø	0.92	Ø	0.35
	S. aureus	1H	Ø	-0.01	Ø	-0.36	Ø	-0.25
		2H	Ø	0.01	↓	1.17	Ø	0.97

	S. epidermidis	1H	Ø	0.06	Ø	0.57	Ø	0.12
		2H	Ø	-0.08	Ø	1.13	Ø	0.61
	C. albicans	1H	Ø	0.26	Ø	-0.17	Ø	-0.11
		2H	Ø	0.07	Ø	0.03	Ø	-0.25

Cord blood (n=10) was stimulated with live microorganisms (*E. coli* 10^4 CFU/ml, *S. aureus* 10^5 CFU/ml, *S. epidermidis* 10^5 CFU/ml, *C. albicans* 10^5 CFU/ml) and simultaneously treated with PTX (200 μM), antimicrobial agents (GEN, VAN or AMB), or combined PTX and antimicrobial agents, and cultured for 1 or 2 hours at 37 ^{o} in 5% CO_2. Mean $\Delta\Delta C_T$ values of samples undergoing different treatment conditions compared to microbial stimulation alone (reference samples). ↑ significant upregulation, ↓ significant downregulation, and ∅ unchanged mRNA expression compared to reference samples, with the number of symbols representing the level of significance ($p \leq 0.05$, $p \leq 0.01$, and $p \leq 0.001$, respectively).

[a] p-values were based on linear mixed model t-tests and adjusted for multiple comparisons employing false discovery rates.

Table S2: Effects of combined PTX and antimicrobial treatment vs PTX or antimicrobial agents alone on microbial-induced gene expression in cord blood

Gene	Microorganism	mRNA expression changes of PTX + antimicrobial agents (Mean ΔΔCT)							
		vs PTX alone [a]				vs antimicrobial agent alone [a]			
		1 hour		2 hours		1 hour		2 hours	
TNF	***E. coli***	Ø	0.34	Ø	0.22	↓	1.53	Ø	0.74
	S. aureus	Ø	-0.38	↓↓	1.53	↓	0.98	↓↓	1.18
	S. epidermidis	Ø	0.11	Ø	0.81	Ø	0.54	Ø	0.35
	C. albicans	Ø	0.09	Ø	-0.12	↓	1.45	↓↓↓	1.22
IL1B	***E. coli***	Ø	0.33	Ø	0.05	Ø	0.43	Ø	-0.33
	S. aureus	Ø	-0.60	↓↓	1.06	Ø	0.53	Ø	0.41
	S. epidermidis	Ø	0.47	Ø	0.86	Ø	0.01	Ø	-0.29
	C. albicans	Ø	-0.23	Ø	-0.21	Ø	0.88	Ø	0.37
IL6	***E. coli***	Ø	0.27	Ø	0.40	Ø	0.01	Ø	-0.39
	S. aureus	Ø	-0.69	↓↓↓	1.44	Ø	-0.40	Ø	-0.05
	S. epidermidis	Ø	-0.03	Ø	1.72	Ø	-1.47	Ø	-1.14
	C. albicans	Ø	-0.79	↑↑	-1.24	Ø	-0.22	Ø	-0.58
IL10	***E. coli***	Ø	0.30	Ø	-0.17	Ø	-0.11	Ø	-0.40
	S. aureus	Ø	-0.18	Ø	0.31	Ø	0.46	Ø	0.34
	S. epidermidis	Ø	-0.35	Ø	-0.51	Ø	-0.06	Ø	-0.44
	C. albicans	Ø	0.47	Ø	-0.26	Ø	0.25	Ø	0.06
TLR2	***E. coli***	Ø	0.04	Ø	-0.41	Ø	0.14	Ø	0.05
	S. aureus	Ø	-0.14	Ø	0.01	Ø	0.18	Ø	0.47
	S. epidermidis	Ø	-0.49	Ø	0.13	Ø	-0.51	Ø	0.13
	C. albicans	Ø	0.08	Ø	-0.10	Ø	0.18	↓	0.50
TLR4	***E. coli***	Ø	-0.42	Ø	-0.42	Ø	0.41	Ø	0.41
	S. aureus	Ø	0.02	Ø	0.02	Ø	0.46	Ø	0.46
	S. epidermidis	Ø	-0.94	Ø	-0.94	Ø	-0.62	Ø	-0.62
	C. albicans	Ø	0.39	Ø	0.39	Ø	1.24	Ø	1.24
RELA	***E. coli***	Ø	-0.09	Ø	-0.28	Ø	0.46	Ø	-0.48
	S. aureus	Ø	-0.10	Ø	0.14	Ø	0.05	Ø	0.11
	S. epidermidis	Ø	-0.52	Ø	-0.36	Ø	-0.42	Ø	-0.44
	C. albicans	Ø	0.47	Ø	-0.07	Ø	0.65	Ø	-0.35
NFKBIA	***E. coli***	Ø	0.04	Ø	-0.05	Ø	0.29	Ø	-0.04
	S. aureus	Ø	-0.16	↓	0.53	Ø	0.32	Ø	0.54
	S. epidermidis	Ø	-0.32	Ø	0.49	Ø	-0.42	Ø	-0.02
	C. albicans	Ø	-0.17	Ø	-0.07	Ø	0.59	Ø	0.21
DUSP1	***E. coli***	Ø	0.22	Ø	0.11	Ø	0.05	Ø	-0.69
	S. aureus	Ø	-0.15	Ø	0.66	Ø	-0.25	Ø	-0.20
	S. epidermidis	Ø	0.03	Ø	0.22	Ø	-0.77	Ø	-0.53
	C. albicans	↓	0.27	Ø	0.30	Ø	-0.18	Ø	-0.46
CASP1	***E. coli***	Ø	0.03	Ø	-0.26	Ø	0.26	Ø	-0.25
	S. aureus	Ø	-0.03	Ø	0.01	Ø	0.47	Ø	0.28
	S. epidermidis	Ø	-0.23	Ø	-0.15	Ø	-0.30	Ø	-0.14
	C. albicans	Ø	0.05	Ø	-0.27	Ø	0.21	Ø	0.03

NLRP3	***E. coli***	Ø	0.16	Ø	0.04	Ø	-0.09	Ø	-0.57
	S. aureus	Ø	-0.24	↓	0.96	Ø	0.11	Ø	-0.20
	S. epidermidis	Ø	0.06	Ø	0.69	Ø	-0.44	Ø	-0.51
	C. albicans	Ø	-0.37	Ø	-0.32	Ø	0.06	Ø	-0.28

Cord blood (n=10) was stimulated with live microorganisms (*E. coli* 10^4 CFU/ml, *S. aureus* 10^5 CFU/ml, *S. epidermidis* 10^5 CFU/ml, *C. albicans* 10^5 CFU/ml) and simultaneously treated with PTX (200 μM), antimicrobial agents (GEN, VAN or AMB), or PTX and antimicrobial agents combined, and cultured for 4 hours at 37 º in 5% CO_2. Mean $\Delta\Delta C_T$ values of samples treated with combined PTX and antimicrobial agents compared to PTX or antimicrobial agents alone (reference samples). ↑ significant upregulation, ↓ significant downregulation, and ∅ unchanged mRNA expression compared to reference samples, with the number of symbols representing the level of significance ($p \leq 0.05$, $p \leq 0.01$, and $p \leq 0.001$, respectively.

[a] p-values were based on linear mixed model t-tests and adjusted for multiple comparisons employing false discovery rates.
